# Stress-induced proteome remodeling at the Golgi–endosome interface

**DOI:** 10.1242/jcs.264535

**Published:** 2026-05-11

**Authors:** Gina Simon, Savina Abraham Pol, Melisa Dendusic, Nina Schulze, Simone Stupia, Michelle Koci, Lana Buzuk, Beatrice Thier, Philine Steinbach, Hai Trinh, Jasmin Schillinger, Sabrina Ninck, Farnusch Kaschani, Markus Kaiser, Michael Ehrmann, Annette Paschen, Doris Hellerschmied

**Affiliations:** ^1^ Department of Mechanistic Cell Biology, Center of Medical Biotechnology, Faculty of Biology, University of Duisburg-Essen, 45141 Essen, Germany; ^2^ Imaging Center Campus Essen, Center of Medical Biotechnology, University of Duisburg-Essen, 45141 Essen, Germany; ^3^ Department of Dermatology, University Hospital Essen, University of Duisburg-Essen, 45147 Essen, Germany; ^4^ Department of Microbiology, Center of Medical Biotechnology, Faculty of Biology, University of Duisburg-Essen, 45141 Essen, Germany; ^5^ Department of Chemical Biology, Center of Medical Biotechnology, Faculty of Biology, University of Duisburg-Essen, 45141 Essen, Germany; ^6^ German Consortium for Translational Cancer Research (DKTK), Partner Site Essen/Düsseldorf, 45147 Essen, Germany

**Keywords:** Golgi stress, Ionophores, Chemical biology, Melanoma, FAM129A, NIBAN1, OSBP inhibitors, RUSH assay

## Abstract

Cellular stress response pathways support cell survival under stress and are often leveraged by cancer cells to gain advantageous traits. How cells respond to Golgi stress is incompletely understood, limiting insights into the role of Golgi stress in cancer. Here, we combined small-molecule stress models and proteomic analyses to elucidate stress-induced changes at the Golgi. Our data establish the depletion of Golgi transport proteins as a common response to different Golgi stressors, including ionophores and oxysterol-binding protein (OSBP) inhibitors. Ionophores further induce *de novo* expression of the stress response protein FAM129A (also known as NIBAN1), which localizes to the remodeled secretory pathway. In a group of melanoma cells, displaying a dedifferentiated epithelial-to-mesenchymal-transition (EMT)-like phenotype, FAM129A is constitutively expressed. In these cells, stress-induced localization of FAM129A to the secretory pathway is achieved by relocalization from the plasma membrane. Collectively, our data highlight the Golgi–endosome interface as a critical hub of the cellular response to Golgi stress and reveal cancer cell-specific effects of this response.

## Teaser

**Highlighted Article:** Ionophore-induced cell stress impairs cargo export from the Golgi, depletes Golgi trafficking proteins and induces FAM129A localization to the secretory pathway.

## INTRODUCTION

The Golgi in mammalian cells consists of layers of membrane-enclosed compartments organized in a polarized fashion into the Golgi stack and laterally linked to form the Golgi ribbon (Klumperman, 2011). It is located at the crossroads of secretory and endocytic pathways. The secretory pathway is responsible for the production of 30% of the cellular proteome. In this capacity, the Golgi is critical for the maturation, sorting and trafficking of secretory and transmembrane cargo proteins, which are initially produced at the endoplasmic reticulum (ER) (Barlowe and Miller, 2013; De Matteis and Luini, 2008; Ramazanov et al., 2021). As part of the endocytic route, the Golgi plays a role in recycling of endosomal and plasma membrane proteins, re-routing them to post-Golgi compartments (Progida and Bakke, 2016). The Golgi is therefore in constant exchange with the ER and the endo-lysosomal system. In the context of this exchange, the distinct identities of the individual organelles need to be maintained, where protein content, lipid composition, lumenal pH and ion composition are distinguishing features.

Cellular health relies on the functionality of individual organelles. Accordingly, organelles have developed stress response pathways, with the Golgi employing different mechanisms to modulate its structure in response to stress (Eisenberg-Lerner et al., 2020; Joshi et al., 2014; Luo et al., 2022). Loss of Golgi structural integrity has served as a read-out revealing Golgi stress as associated with neurodegenerative diseases and certain types of cancer (Makhoul et al., 2019). Moreover, secretory pathway stressors have been explored as anti-cancer drugs (Burgett et al., 2011; Cigler et al., 2025; Marjanovic et al., 2023) focusing on vulnerabilities specific to cancer cells undergoing epithelial-to-mesenchymal transition (EMT) (Feng et al., 2014; Gupta et al., 2009; Tan et al., 2025). To understand its role in cellular homeostasis and disease processes, a better understanding of the Golgi stress response is required.

Small-molecule stressors that interfere with Golgi functionality are applied as tools to study how the Golgi manages stress and how it can restore homeostasis. The ionophores nigericin, monensin and salinomycin reversibly permeabilize membranes to H^+^, K^+^ and Na^+^ ions, inducing a remodeling of the Golgi and the endosomal system in mammalian cells (Bachert et al., 2001; Marjanovic et al., 2023; Mollenhauer et al., 1990; Podinovskaia et al., 2021; Zhang et al., 2023). Based on studying model cargo proteins, ionophores are known to block protein secretion and endosome-to-Golgi trafficking (Bachert et al., 2001; Serebrenik et al., 2018; Zhang et al., 2023). Another strategy to induce Golgi stress is the use of oxysterol-binding protein (OSBP) inhibitors, which interfere with the exchange of cholesterol and phosphatidylinositol-4-phosphate [PI(4)P] between Golgi and ER (Mesmin et al., 2017). OSBP inhibitors thereby interfere with lipid homeostasis and induce the depletion of Golgi proteins (Cigler et al., 2025; He et al., 2025). Recent mechanistic studies have further reported ubiquitin-dependent remodeling of the Golgi matrix (Eisenberg-Lerner et al., 2020; Luo et al., 2022) and Golgiphagy (Hickey et al., 2023; Kitta et al., 2024; Yang et al., 2025) in response to stress, as well as the activation of transcriptional Golgi stress response pathways (Oku et al., 2011; Reiling et al., 2013; Serebrenik et al., 2018).

FAM129A (also known as NIBAN1) is a stress response gene that is expressed downstream of the transcription factor ATF4 in response to ER and Golgi stress (Pallmann et al., 2019; Serebrenik et al., 2018). It belongs to a three-member protein family including FAM129B (also known as MINERVA and NIBAN2), which promotes melanoma invasion (Old et al., 2009), and FAM129C (also known as NIBAN3). FAM129A expression is elevated in several cancers, including renal carcinoma (Feng et al., 2019), prostate cancer (Pallmann et al., 2019) and thyroid carcinoma (Carvalheira et al., 2015; Nozima et al., 2019), independent of external stressors. Knock-down of FAM129A in thyroid cancer cells reduces cell migration (Carvalheira et al., 2015), suggesting a role in cancer cell motility. Under stress conditions, a role for FAM129A in regulating protein translation has been described (Pallmann et al., 2019; Sun et al., 2007).

Here we performed an in-depth analysis of the cellular response to Golgi stress, focusing on proteomic changes in response to ionophores. We establish the depletion of Golgi transport proteins as a shared, early phenotype in response to different Golgi stressors. The stress response protein FAM129A localizes to the remodeled secretory pathway upon ionophore-induced stress. We additionally identify FAM129A as constitutively expressed in dedifferentiated melanoma cells, where it is enriched at the plasma membrane close to actin-organizing centers. In those cells, ionophore-induced stress sequesters the constitutively expressed FAM129A away from the plasma membrane to the Golgi–endosome interface, highlighting cancer cell-specific effects of the cellular stress response.

## RESULTS AND DISCUSSION

### Stress-induced secretory pathway remodeling

**Fig. 1.**
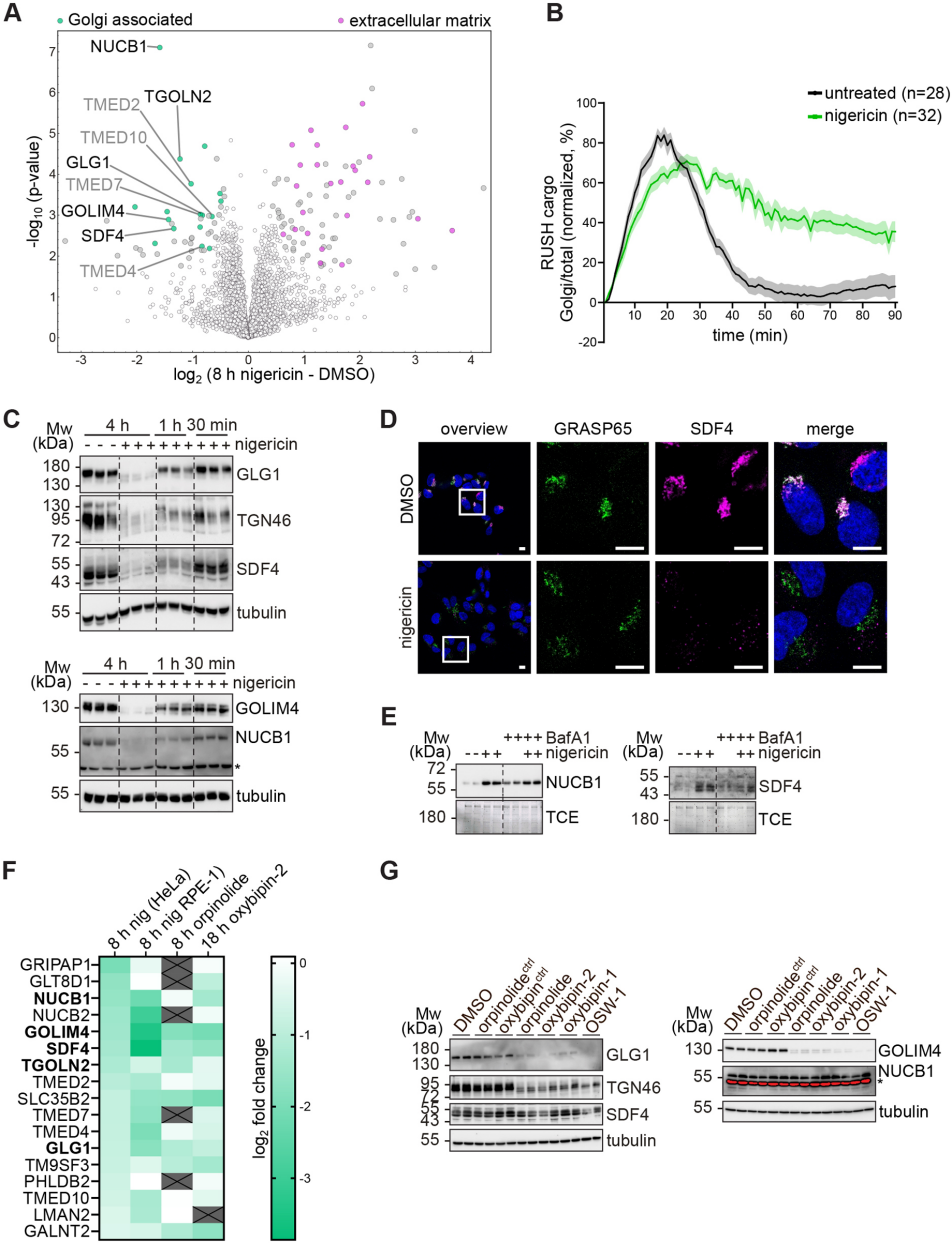
**Nigericin-induced proteomic changes.** (A) Volcano plot representing quantitative proteomics data obtained from HeLa cells treated with 1 µM nigericin. (B) RUSH assay kinetics of HeLa cells expressing Str-li-IRES-VSVG-SBP-EGFP and ST6Gal1–mCherry. Cells were treated with 1 µM nigericin 4 h prior to biotin addition or left untreated. Quantitative data depicts combined mean±s.e.m. of single cells for each condition. Data obtained from two independent experiments. (C) Western blot analysis of RPE1 cells upon 1 µM nigericin treatment for the indicated times. Blots shown are representative of three experiments. (D) Representative confocal sections depicting immunofluorescence of SDF4 (magenta) and the Golgi marker GRASP65 (green) in RPE1 cells treated with 1 µM nigericin for 30 min. Nuclei were stained with Hoechst 33342 (blue). Boxes indicate regions shown in the enlarged images. Scale bars: 10 µm. Image shown are representative of three experiments. (E) Western blot analysis of conditioned media of RPE1 cells treated with 1 µM nigericin and/or 0.1 µM BafA1 for 90 min. Blots shown are representative of three experiments. (F) Heat map comparing the decrease of 17 Golgi proteins by 1 µM nigericin treatment (nig) in HeLa or RPE1 cells to 1 µM oxybipin-2 in HeLa cells (He et al., 2025) or 1 µM orpinolide in KBM7 cells (Cigler et al., 2025). (G) Western blot analysis of RPE1 cells upon 1 µM compound treatment, as indicated, for 8 h. Blots shown are representative of two experiments. Asterisks in C and G mark non-specific bands.

Ionophores are widely used as Golgi stressors due to their established effect in blocking protein secretion and disrupting Golgi structure (Bui et al., 2023; Eisenberg-Lerner et al., 2020; Oku et al., 2011; Serebrenik et al., 2018). Here, we aimed to explore the associated proteomic changes. We analyzed a quantitative mass spectrometry (MS) dataset obtained from HeLa cells treated with 1 µM nigericin for 1, 4 or 8 h. In total we identified 4411 proteins, with the levels of 146 of them significantly changed at the 8 h treatment time point [false discovery rate (FDR)≤0.05, artificial within-groups variance (s0) of 0.1; Fig. 1A]. These 146 proteins showed a similar trend at the earlier treatment time points (Table S1, Fig. S1A,B). We found that the set of proteins with increased levels is enriched in extracellular matrix components, including collagens, likely reflecting the impaired secretory capacity of the nigericin-treated cells (Table S1; Fig. 1A). To understand how nigericin-induced Golgi stress impacts anterograde trafficking through the Golgi, we monitored transport kinetics using the Retention Using Selective Hooks (RUSH) system (Boncompain et al., 2012) (Fig. 1B; Movies 1 and 2). The plasma membrane-targeted RUSH cargo VSVG–SBP–EGFP is retained at the ER by the ER-resident streptavidin hook Ii-Str and is released by the addition of biotin. Under control conditions, the RUSH cargo reached the Golgi, as defined by the marker ST6Gal1–mCherry, within 15–22 min of biotin addition. By 50 min after biotin addition, the majority of the cargo had passed through the Golgi (Fig. 1B). Pre-treatment of the cells with nigericin for 4 h caused a delay in export from the Golgi, where even after 90 min of biotin addition, a large fraction of the RUSH cargo still localized to the Golgi (Fig. 1B). We then analyzed proteins with reduced cellular levels upon nigericin treatment (55 proteins) and identified 17 proteins that localize to the Golgi and/or facilitate Golgi transport processes (Table S1). We followed up on the response of five proteins [NUCB1, SDF4 (also known as CAB45), TGN46 (also known as TGOLN2), GLG1, GOLIM4 (also known as GPP130)] with an involvement in (post-)Golgi trafficking. The soluble Golgi lumenal proteins NUCB1 and SDF4 facilitate the export of secretory cargo from the Golgi (Blank and von Blume, 2017; Pacheco-Fernandez et al., 2020; Ramazanov et al., 2021). The transmembrane proteins TGN46, GLG1 and GOLIM4 traffic through post-Golgi compartments with a steady-state Golgi localization. Specifically, GOLIM4 is involved in endosome-to-Golgi trafficking (Forrester et al., 2020; Natarajan and Linstedt, 2004), and TGN46 is important for the sorting and export of secretory proteins from the trans-Golgi network in CARriers of the trans-Golgi network to the cell Surface (CARTS) (Lujan et al., 2024; Wakana et al., 2012).

We performed this more detailed analysis in RPE1 cells, a non-cancer cell line with a large Golgi, suitable for microscopy studies. The levels of all five transport proteins robustly decreased upon nigericin and monensin treatment at short treatment time points (30 min to 4 h) (Fig. 1C; Fig. S1C). We also corroborated the observed changes in a quantitative MS experiment in RPE1 cells following 8 h nigericin treatment (Fig. S1D, Table S1). These MS data matched the data obtained from HeLa cells (Fig. S1E). We next investigated how nigericin affects the steady-state localization of the transport chaperones SDF4 and NUCB1. Both proteins were observed to localize to the Golgi, as outlined by the Golgi matrix proteins GRASP65 (also known as GORASP1) or p230 (also known as GOLGA4) (Fig. 1D; Fig. S2A). Treatment with nigericin caused an acute redistribution of SDF4 and NUCB1 proteins away from the Golgi, as defined by the Golgi matrix proteins, towards vesicular structures (Fig. 1D; Fig. S2A). Under these conditions we detected SDF4 and NUCB1 in conditioned culture medium of RPE1 cells (Fig. 1E). SDF4 and NUCB1 are Ca^2+^-binding proteins, and previous work by von Blume and colleagues has demonstrated that Golgi retention of SDF4 is Ca^2+^ dependent (von Blume et al., 2012). We reasoned that the nigericin-induced change in pH (Zhang et al., 2023) and ion composition of the Golgi lumen impaired the retention of SDF4 and NUCB1 at the Golgi. Consistently, a similar effect was observed for the treatment with bafilomycin A1 (BafA1), a V-ATPase inhibitor affecting the pH of lysosomes and the Golgi (Fig. 1E) (Liu et al., 2023; Llopis et al., 1998). These data suggest that the acute release of SDF4 and NUCB1 from the Golgi and into the culture medium is a shared response to stressors that affect the pH of the Golgi lumen.

In addition to increasing the pH of the Golgi lumen, nigericin also causes an increase in the levels of the signaling lipid PI(4)P in the Golgi membrane and blocks endosome-to-Golgi trafficking (Zhang et al., 2023). These phenotypic effects are shared with Golgi stressors that target OSBP (Cigler et al., 2025; He et al., 2025; Mesmin et al., 2017). Moreover, the OSBP inhibitors orpinolide and oxybipins cause a decrease of Golgi proteins (Cigler et al., 2025; He et al., 2025). We compared our dataset of proteins significantly decreased after 8 h nigericin treatment in HeLa cells to the dataset obtained from nigericin-treated RPE1 cells and published proteomics datasets obtained upon orpinolide or oxybipin-2 treatment (Cigler et al., 2025; He et al., 2025). Although the time points and cell lines of the datasets do not exactly match, we found that Golgi proteins that decreased in response to nigericin were also identified as decreased upon OSBP inhibition (Fig. 1F). For a direct comparison, we analyzed the effects of orpinolide, oxybipin-1, oxybipin-2 and OSW-1 (a member of a third OSBP inhibitor class, the ORPphilins; Burgett et al., 2011) on the five select proteins previously analyzed upon nigericin and monensin treatment in RPE1 cells. The inactive orpinolide (orpinolide^crtl^) and *N*-methyl cholenamide (oxybipin^ctrl^) served as controls for the OSBP-targeting compounds (Cigler et al., 2025; He et al., 2025). The levels of NUCB1 and SDF4, which we propose to react to acute changes in pH upon nigericin and BafA1 treatment, were reduced only at extended treatment times of 18 h (Fig. S2B). Notably, we observed a robust decrease of the transmembrane proteins TGN46, GOLIM4 and GLG1 after 8 h (Fig. 1G). These data show that the depletion of the Golgi transport proteins TGN46, GOLIM4 and GLG1 is a shared response to ionophores (nigericin and monensin) and different OSBP inhibitors (orpinolide, oxybipins, OSW-1).

Taken together, our findings demonstrate an extended Golgi residence time of secretory cargo upon nigericin-induced stress and highlight the depletion of post-Golgi trafficking proteins as a characteristic early event in the cellular response to Golgi stress.

### FAM129A responds to secretory pathway stress

In addition to the depletion of Golgi transport proteins, nigericin induces a transcriptional stress response (Serebrenik et al., 2018), including several target genes of the transcription factor ATF4. Consistently, nigericin induces the expression of ATF4 in HeLa cells, following 4 h of treatment. The kinetics of ATF4 expression are slower than with the established ER stressor thapsigargin but show a comparable level of induction (Fig. S3A). To understand how the transcriptional stress response relates to the remodeling of the secretory pathway, we focused on FAM129A, an ATF4 target gene that is induced by nigericin and proteotoxic Golgi stress, but also ER stress (Pallmann et al., 2019; Serebrenik et al., 2018).

**Fig. 2.**
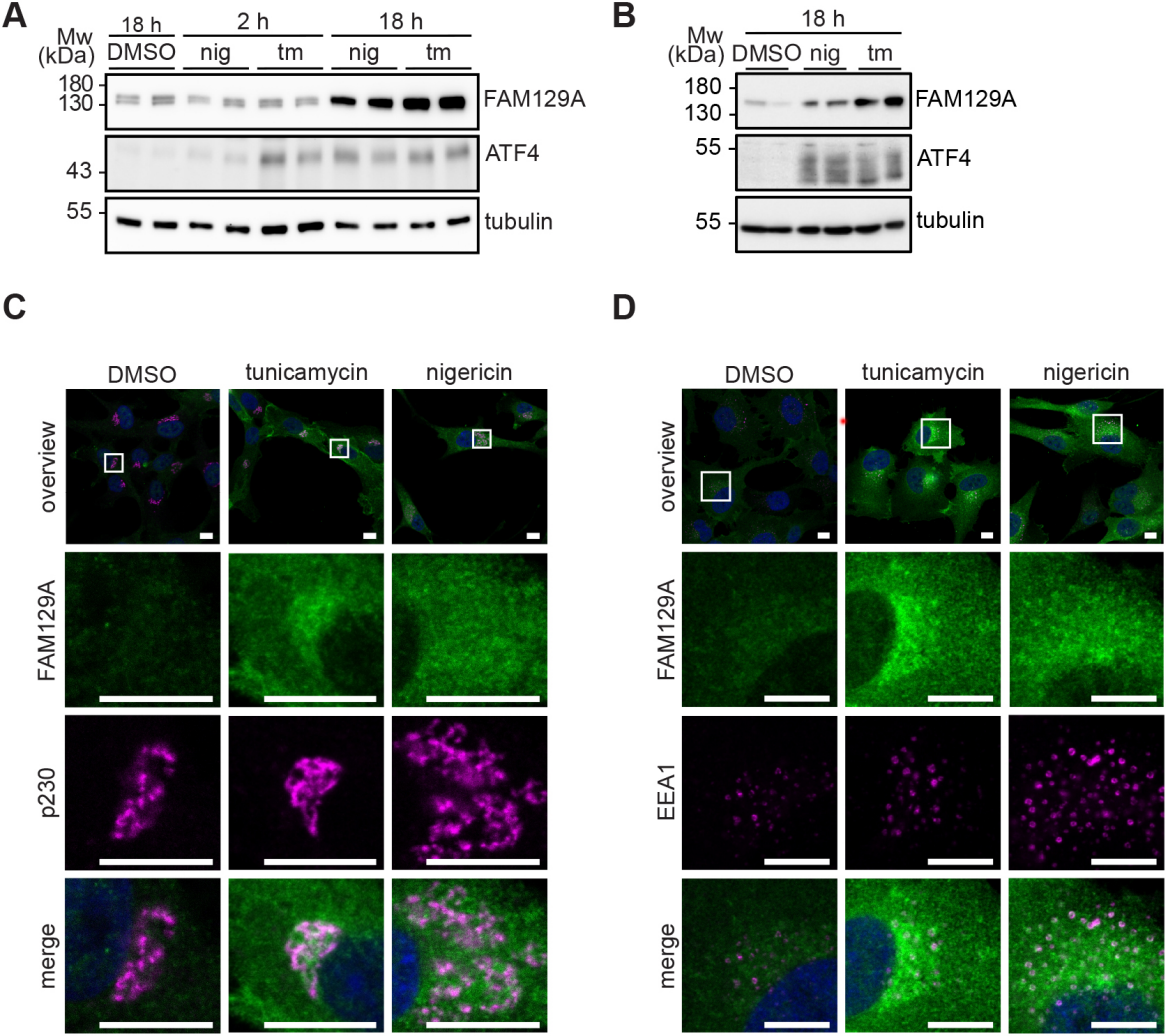
**Stress-induced FAM129A.** (A,B) Western blot analysis of (A) HeLa and (B) RPE1 cells treated with 1 µM nigericin (nig) or 5 µg/ml tunicamycin (tm) for the indicated times. Blots shown are representative of three experiments. (C,D) Representative confocal sections depicting immunofluorescence of FAM129A (green) and either (C) the Golgi marker p230 (magenta) or (D) the endosome marker EEA1 (magenta) in RPE1 cells treated as in B. Nuclei were stained with Hoechst 33342 (blue). Boxes indicate regions shown in the enlarged images. Scale bars: 10 µm. Images of FAM129A localization are representative of four experimental repeats.

**Fig. 3.**
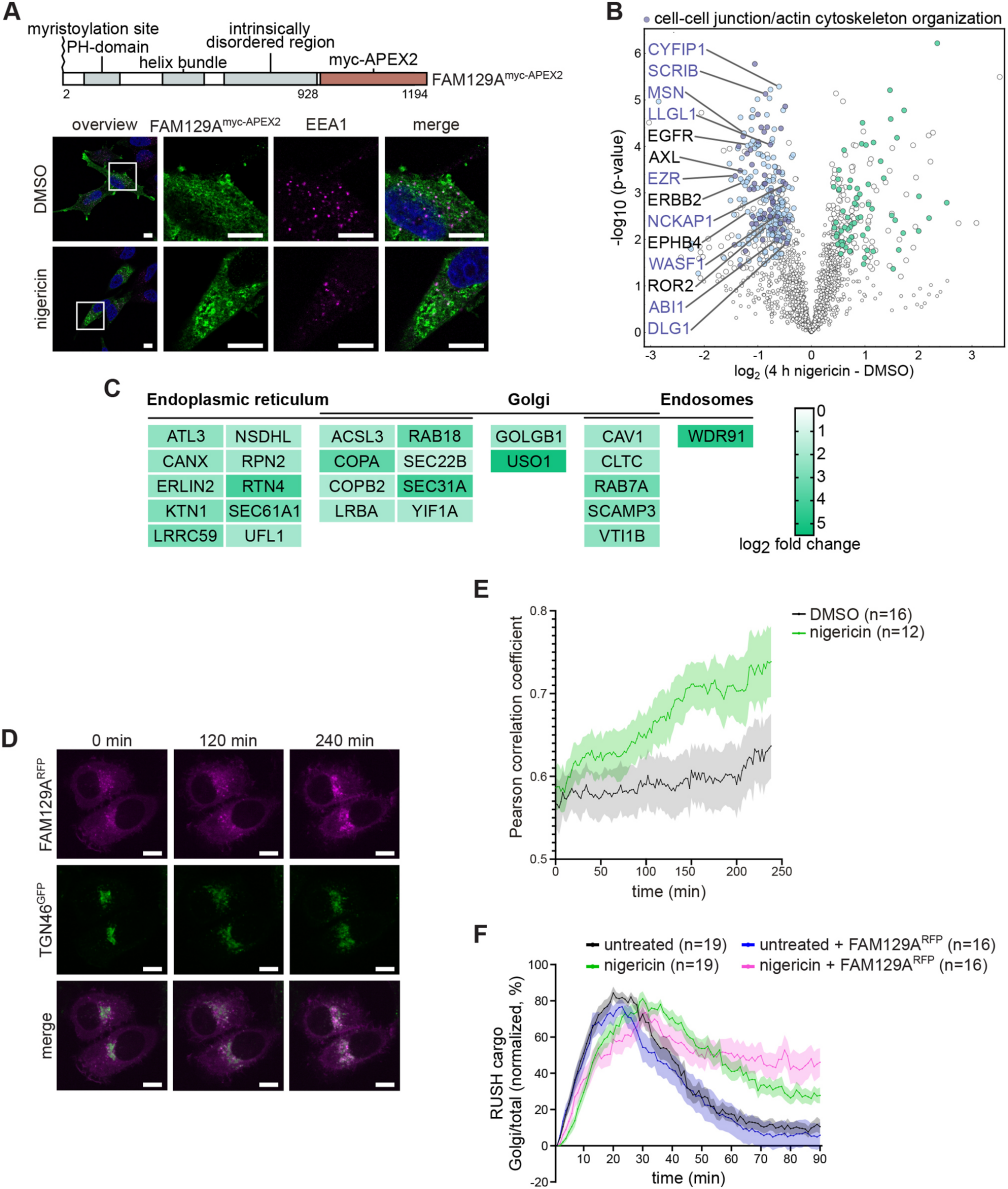
**FAM129A localizes to the secretory pathway during stress.** (A) Top: diagram showing the domain structure of FAM129A^myc–APEX^, with amino acid positions indicated. Bottom: representative confocal sections depicting immunofluorescence of HeLa FAM129A^myc–APEX^ cells induced with 100 ng/ml doxycycline for 22 h and treated with 1 µM nigericin for the last 4 h. Cells were immunolabeled to detect FAM129A (green) and EEA1 (magenta). Nuclei were stained with Hoechst 33342 (blue). Boxes indicate regions shown in the enlarged images. Scale bars: 10 µm. Images of FAM129A^myc–APEX2^ localization are representative of three experimental repeats. (B) Volcano plot of proteins biotinylated by FAM129A^myc–APEX^ upon treatment with 1 µM nigericin or DMSO control. Significant hits excluding potential contaminants are colored. (C) Heat map representing secretory pathway-localized hits in proximity to FAM129A^myc–APEX^ specifically upon nigericin treatment. (D) Individual time points of live-cell imaging of HeLa Kyoto cells expressing FAM129A^RFP^ and TGN46^GFP^ during 4 h of treatment with 1 µM nigericin. Scale bars: 10 µm. (E) Pearson correlation coefficient (mean±s.e.m.) of cells treated and imaged as shown in D. All cells recorded in one experiment. (F) RUSH assay kinetics of HeLa Kyoto cells expressing Str-li-IRES-VSVG-SBP-EGFP and GRASP65–SNAP with or without FAM129A^RFP^. Cells were pre-treated with 1 µM nigericin for 4 h prior to biotin addition or left untreated. Quantitative data depicts combined mean±s.e.m. of single cells for each condition. All cells recorded in one experiment.

In HeLa and RPE1 cells, we observed an increase in FAM129A protein levels following 18 h of treatment with nigericin or the ER stressor tunicamycin (Fig. 2A,B). These data are consistent with a previous report by Paellmann et al. classifying FAM129A as a late ATF4 response gene (Pallmann et al., 2019). We then assessed the localization of stress-induced FAM129A. In tunicamycin-treated RPE1 cells, FAM129A was enriched at a juxtanuclear region near the Golgi, as outlined by the trans-Golgi marker p230 (Fig. 2C). Nigericin treatment induces Golgi fragmentation and transitioning of Golgi membranes into endosomes, leading to their enlargement (Podinovskaia et al., 2021; Zhang et al., 2023). These phenotypes were visualized with the Golgi marker p230 and the early endosome marker EEA1, respectively. Notably, in nigericin-treated cells, we observed an enrichment of FAM129A at EEA1-positive vesicles (Fig. 2D). We additionally characterized the localization of a FAM129A overexpression construct carrying a C-terminal myc–APEX2 tag (FAM129A^myc–APEX2^). Interestingly, in HeLa cells, FAM129A^myc–APEX2^ localized to the ER and was enriched at the plasma membrane at cell protrusions resembling actin-based lamellipodia with membrane ruffles (Fig. 3A), consistent with a previous report (Pallmann et al., 2019). A 4 h nigericin treatment led to a reduction of the signal at the plasma membrane and an increase at larger vesicular structures, indicating a stress-induced relocalization (Fig. 3A). Of note, FAM129A^myc–APEX2^ expression levels were comparable to nigericin- and tunicamycin-induced protein levels, and the total levels of FAM129A^myc–APEX2^ did not change during the 4 h nigericin treatment (Fig. S3B,C). To precisely map the surroundings of FAM129A^myc–APEX2^, we performed an APEX2-based proximity biotinylation experiment, comparing stress (nigericin) and non-stress (DMSO) conditions. Following biotinylation and streptavidin pull-down, the elution fractions were analyzed by MS (Fig. 3B; Table S2). In total, we detected 1.635 proteins, with 527 proteins that significantly changed in abundance upon streptavidin pull-down (FDR≤0.01, s0 of 0.1). To ensure robust and reliable data analysis, the experiment was performed twice with highly reproducible results (Table S2), and we excluded common contaminants typically identified in streptavidin pull-down studies from further analysis (Mellacheruvu et al., 2013).

The set of proteins showing decreased proximity to FAM129A^myc–APEX^ upon nigericin treatment was enriched in plasma membrane-localized proteins, particularly those forming part of the Gene Ontology (GO) terms ‘cell–cell junction’ and ‘actin cytoskeleton organization’ (Fig. 3B; Table S2). Conversely, this suggests that these proteins are in close proximity to FAM129A specifically under non-stress conditions, consistent with our imaging data (Fig. 3A). The dataset includes receptor tyrosine kinases, EGFR, ERBB2, EPHB4, ROR2 and AXL with a role in growth factor signaling. We further detected all three subunits of the Scribble polarity complex (SCRIB, LLGL1 and DLG1), a key regulator of cell polarity and directional cell migration (Stephens et al., 2018). Close proximity to proteins that regulate the reorganization of the actin cytoskeleton matches the enrichment of FAM129A at structures resembling lamellipodia and ruffles (Fig. 3A). Specifically, ezrin (EZR) and moesin (MSN) link the actin cytoskeleton to the plasma membrane and control the integrity of the cell cortex (Clucas and Valderrama, 2014). We further detected four of the five subunits of the WAVE regulatory complex [WASF1 (also known as WAVE1), CYFIP1 (also known as SRA1), NCKAP1 (also known as NAP1) and ABI1], a critical regulator of the actin cytoskeleton in the leading edge of migrating cells (Wu et al., 2025). These putative interactors at the plasma membrane are in line with a described role for FAM129A in cancer cell migration (Carvalheira et al., 2015; Yuki et al., 2015). Under nigericin stress conditions, 26 of 68 proteins detected in proximity to FAM129A^myc–APEX^ suggest its localization to the secretory pathway (Fig. 3B,C; Table S2). Specifically, we found proteins with a role in Golgi trafficking at the ER–Golgi and at the Golgi–endosome interface, consistent with imaging data of the stress-induced FAM129A protein (Fig. 3B,C). We then performed live-cell imaging to visualize the nigericin-induced change in localization of FAM129A over time. In line with the proximity labeling data, we found that nigericin increased the colocalization of RFP-tagged FAM129A (FAM129A^RFP^) with GFP-tagged TGN46 (TGN46^GFP^), a marker for the rearranged trans-Golgi network (Fig. 3D,E). We then explored whether FAM129A would affect protein secretion using the RUSH assay. We found that the expression of FAM129A^RFP^ and its nigericin-induced change in localization did not affect the secretory capacity of the cell (Fig. 3F).

Taken together, our data support that constitutively expressed FAM129A predominantly localizes to the plasma membrane in proximity of actin regulatory proteins. Under nigericin stress conditions, it localizes to the remodeled secretory pathway. With differences in response time, this stress-dependent localization of FAM129A might be achieved by the synthesis of new protein (in RPE1 cells) or the relocalization of a pre-existing pool of protein (overexpression of FAM129A in HeLa cells). The expression of FAM129A and its nigericin-induced enrichment at the secretory pathway did not affect secretory protein transport.

### FAM129A during secretory pathway stress in melanoma

**Fig. 4.**
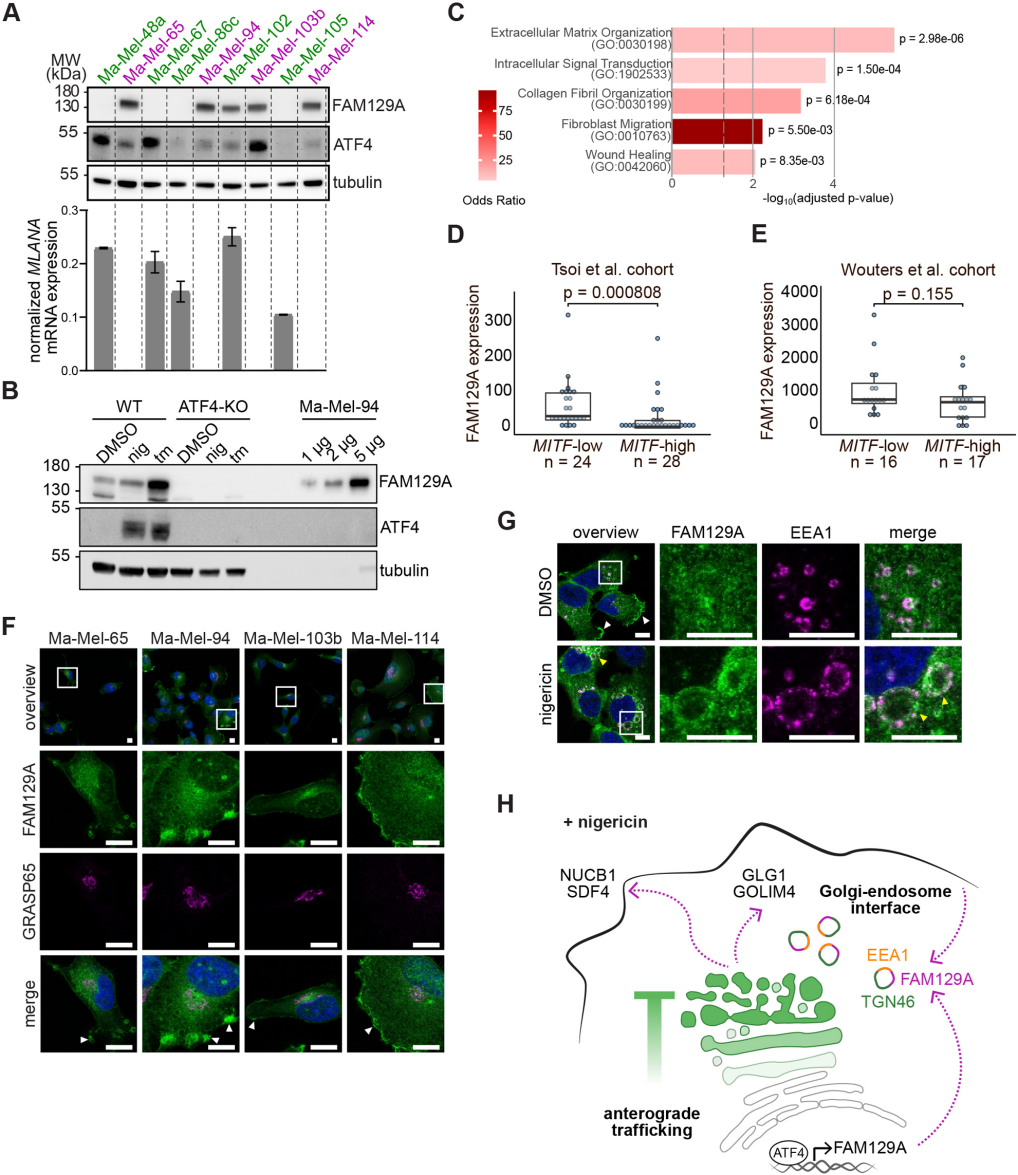
**FAM129A in melanoma.** (A) Top: western blot analysis of FAM129A in melanoma cell lines (magenta, dedifferentiated cell line; green, differentiated cell line). Bottom: differentiation state was determined by qPCR quantifying *MLANA* expression (normalized to *RPL13A* expression; mean±s.e.m., *n*=2). (B) Western blot analysis of FAM129A and ATF4 in Ma-Mel-61a wild-type (WT) and ATF4 KO cells treated with 1 µM nigericin (nig) or 5 µg/ml tunicamycin (tm) for 18 h (25 µg total protein applied). The indicated loadings of Ma-Mel-94 lysate are shown for comparison. Blots shown are representative of three experiments. (C) GO enrichment analysis of the top 100 genes positively correlating with *FAM129A* expression in melanoma cell lines from Tsoi et al. (2018). The vertical dashed line indicates an adjusted *P*-value threshold of <0.05. Color scale represents the odds ratio. (D,E) *FAM129A* expression in *MITF*-low and *MITF*-high melanoma cell lines from (D) Tsoi et al. (2018) and (E) Wouters et al. (2020) datasets. *P*-value calculated with two-sided Wilcoxon rank-sum test. Boxplots depict the median (horizontal line), interquartile range (box) and the most extreme values within 1.5× the interquartile range (whiskers). (F,G) Confocal imaging of FAM129A localization in melanoma cell lines. (F) Representative confocal sections depicting immunofluorescence of FAM129A (green) and the Golgi marker GRASP65 (magenta) in the indicated dedifferentiated melanoma cell lines. (G) Representative confocal sections depicting immunofluorescence of FAM129A (green) and EEA1 (magenta) in Ma-Mel-94 cells treated with 1 µM nigericin for 4 h. Nuclei were stained with Hoechst 33342 (blue). Boxes indicate regions shown in the enlarged images. White and yellow arrows indicate enrichment of FAM129A at the plasma membrane or endomembranes, respectively. Scale bars: 10 µm. Images are representative of two experimental repeats. (H) Model of stress-induced remodeling of the secretory pathway.

Whereas we induced high FAM129A levels by overexpression in HeLa cells, previous studies have demonstrated constitutively high FAM129A expression in different cancer cell lines (Ayesha et al., 2022; Cao et al., 2022; Feng et al., 2019; Nozima et al., 2019; Wang et al., 2021). To study Golgi stress and FAM129A expression in the context of cancer, we selected melanoma as an understudied model. Analyses of FAM129A protein levels by western blotting revealed aberrant constitutive expression in five of nine patient-derived melanoma cell lines (Fig. 4A). Notably, melanoma cell lines with high FAM129A levels were classified as dedifferentiated (Fig. 4A), based on the differentiation marker Melan-A (also known as *MLANA*), a transcriptional target of MITF (Fig. 4A; Fig. S4A). In the differentiated melanoma cell line Ma-Mel-61a (Thier et al., 2022), which has comparatively low basal FAM129A levels (Fig. 4B), we observed that FAM129A expression was induced in response to Golgi and ER stress (Fig. 4B; Fig. S4B). Using a corresponding Ma-Mel-61a ATF4 knock-out (KO) cell line, we found that this stress-induced FAM129A expression is largely dependent on ATF4 (Fig. 4B; Fig. S4B). Strikingly, constitutive FAM129A expression levels in dedifferentiated melanoma cell lines could not be predicted based on the ATF4 protein levels (Fig. 4A), suggesting an involvement of additional regulatory mechanisms. To gain insights into cellular programs associated with constitutive FAM129A expression, we analyzed a public transcriptomic dataset derived from 53 human melanoma cell lines (Tsoi et al., 2018). These analyses revealed association of *FAM129A* with genes involved in extracellular matrix organization and positive regulation of fibroblast migration (Fig. 4C). Notably, it is well established that melanoma cells with enhanced migratory and invasive capacity show a rather dedifferentiated phenotype, characterized by downregulation of the lineage differentiation program and its transcriptional regulator MITF (Rambow et al., 2019). To this end, analyses of two public datasets (Tsoi et al., 2018; Wouters et al., 2020) revealed increased *FAM129A* mRNA levels in dedifferentiated *MITF*-low melanoma cells compared to differentiated *MITF*-high cells (Fig. 4D,E). Moreover, we analyzed single-cell RNA sequencing data and found higher levels of FAM129A in dedifferentiated (mesenchymal-like) cells compared to differentiated (melanocytic) melanoma cells (Fig. S4C).

In these dedifferentiated melanoma cell lines, we observed a strong FAM129A signal at the plasma membrane with an enrichment at lamellipodia-like projections and membrane ruffles (Fig. 4F). This matches the localization and the proximome of overexpressed FAM129A in HeLa cells (Fig. 3A,B) and supports a role for FAM129A at structures that drive cell migration. We then treated Ma-Mel-94 cells with nigericin to induce Golgi stress and monitored an established stress phenotype – the Golgi-to-endosome shift of TGN46 (Podinovskaia et al., 2021; Zhang et al., 2023). We observed that in Ma-Mel-94 cells, TGN46 also shifts to EEA1-positive endosomes in response to nigericin (Fig. S4D). Notably, following nigericin treatment for 4 h, the FAM129A signal was reduced at the plasma membrane, while enriched at enlarged EEA1-positive structures (Fig. 4G; Fig. S4E), consistent with the localization of stress-induced FAM129A and data obtained for nigericin-treated FAM129A-overexpressing HeLa cells. This phenotype was not observed upon treatment with OSBP inhibitors (Fig. S4F), suggesting an ionophore-specific effect.

Taken together, our data suggest that constitutive FAM129A expression in melanoma cells is associated with a dedifferentiated phenotype. In dedifferentiated melanoma cells, the nigericin-induced localization of FAM129A to the Golgi–endosome interface therefore constitutes an early event.

### Principles of secretory pathway remodeling

In this study, using small-molecule Golgi stressors in cell culture models, we derive common principles of stress-induced secretory pathway remodeling (Fig. 4H). Our quantitative proteomics data, together with previously published datasets, establish the depletion of Golgi transport proteins as a common early event in response to stress. We suggest that determining the levels of these select Golgi proteins should be included in the characterization of secretory pathway stressors. The Golgi lumenal proteins SDF4 and NUCB1 are secreted, an effect that is shared by stressors that increase the pH of the Golgi lumen. Ionophores and OSBP inhibitors share their effect on depleting TGN46, GOLIM4 and GLG1. Mechanistically, previous work has shown that in response to ionophores, TGN46 and GOLIM4 transition into endosomes (Linstedt et al., 1997; Podinovskaia et al., 2021; Starr et al., 2007; Tewari et al., 2015; Zhang et al., 2023). As a wider-ranging phenomenon affecting multiple Golgi proteins (including TGN46, GOLIM4 and GLG1), the Golgi-to-endosome shift has been shown by spatial proteomics in response to stress induced by starvation and BafA1 treatment (Schessner et al., 2023). The relocalization of Golgi transmembrane proteins to endosomes might be the sole consequence of impaired or deregulated transport processes; however, the enlarged endosomes that acquire Golgi proteins might serve as a storage and sorting center, allowing the Golgi and endosomes to reform as organelles with distinct identities upon stress relief, when transport processes resume. Equally, the lysosomal degradation pathway can be accessed from endosomes and serve to degrade Golgi proteins affected by stress. Our proteomics data revealed that nigericin, similar to oxybipin-2 (He et al., 2025), also induces a decrease of transmembrane emp24 domain-containing proteins (TMED2, TMED4, TMED7 and TMED10; Fig. 1A,F) with a role in ER–Golgi trafficking, and it will be interesting to follow up on their depletion in future work. In addition to secretion, localization to endosomes and lysosomal degradation, the stress-induced proteasomal degradation of a Golgi-associated protein has also been reported (Eisenberg-Lerner et al., 2020). Finally, FAM129A, which is an ATF4 target gene that peripherally associates with cellular membranes at their cytosolic side, localizes to the Golgi–endosome interface under nigericin stress conditions. This introduces the concept of recruiting transcriptional stress response targets to the remodeled secretory pathway.

### The stress response protein FAM129A in melanoma

The stress response protein FAM129A, when constitutively expressed in melanoma or overexpressed in HeLa cells, is enriched at the plasma membrane in proximity to regulators of the actin cytoskeleton, aligning with its described role in cancer cell motility (Carvalheira et al., 2015; Yuki et al., 2015). Notably, a role for its homolog FAM129B in growth factor signaling and cell motility in melanoma is well established (Conrad et al., 2013; Old et al., 2009). We speculate that ionophores impair the cancer-specific function of FAM129A, given that nigericin treatment strongly reduces the proximity of FAM129A to receptor tyrosine kinases and actin-organizing proteins. We thereby highlight an example of how cancer cells – with their markedly different proteome, including upregulated stress response targets – are affected differently by Golgi stressors.

## MATERIALS AND METHODS

### Reagents

Orpinolide (W7) and the corresponding control compound orpinolide^ctrl^ (W7-C) were a kind gift from Herbert Waldmann (Max-Planck Institute of Molecular Physiology, Dortmund, Germany; Cigler et al., 2025). Oxybipin-1, oxybipin-2 and the corresponding *N*-methyl cholenamide control were a kind gift from Luca Laraia (Technical University of Denmark, Kongens Lyngby, Denmark; He et al., 2025). All other compounds were purchased from commercial suppliers: nigericin (Merck Millipore, 481990), monensin (Calbiochem, 475895), OSW-1 (MedChemExpress, #HY-101213), tunicamycin (Sigma-Aldrich, #T7765), thapsigargin (Sigma-Aldrich, #T9033), bafilomyin A1 (Alfa Aesar, #J61835), doxycycline hyclate (Sigma-Aldrich, D9891). For antibodies see Tables S3 and S4.

### DNA constructs and molecular cloning

All primers used for the generation of DNA constructs are listed in Table S5. FAM129A was amplified from HEK293 cDNA and initially cloned into pcDNA3 containing mRFP–FLAG, previously described in Serebrenik et al. (Serebrenik et al., 2018). To generate FAM129A^myc–APEX2^ in pcDNA5/FRT/TO, FAM129A was amplified from the FAM129A–mRFP–FLAG construct and first assembled in a pcDNA3 vector containing myc–APEX2 (a gift from Hemmo Meyer, University of Duisburg-Essen, Center of Medical Biotechnology, Essen, Germany) using the NEB Hifi Builder mix (New England Biolabs) according to the manufacturer's instructions. The FAM129A–myc–APEX2 sequence was then amplified and assembled into a pcDNA5/FRT/TO vector (Invitrogen) using the NEB Hifi Builder mix (New England Biolabs). To generate GRASP65–SNAP in pcDNA3, GRASP65 was amplified from cDNA and SNAP was amplified from SNAP–EGFP in pcDNA5/FRT/TO (Abraham Pol et al., 2024). The two PCR products were introduced into pcDNA3 (Invitrogen) using the NEB Hifi Builder mix. Str-Ii_IRES_VSVG-SBP-EGFP in pIRESneo3 was a gift from Amy Hudson (Medical College of Wisconsin, Milwaukee, WI, USA), ST6GAL1–mCherry in pCMV3 and TGN46–EGFP in pEGFP-N1 were a gift from Felix Campelo (Institut de Ciencies Fotoniques, The Barcelona Institute of Science and Technology, Barcelona, Spain; Lujan et al., 2024). Plasmids are available from the authors upon request.

### Cell culture and small-molecule stress treatments

Patient-derived melanoma cell lines were provided by the Westdeutsche Biobank Essen (WBE/SCABIO, University Hospital Essen, University of Duisburg-Essen, Essen, Germany; approval no. SCABIO_114715) and cultured in RPMI (Thermo Fisher Scientific, #21875-034) supplemented with 10% fetal bovine serum (FBS; Thermo Fisher Scientific), 100 U/ml penicillin and 100 μg/ml streptomycin (Thermo Fisher Scientific #15140122). RPE1 cells (ATCC) were cultured in DMEM/F12 (Thermo Fisher Scientific, #31331028) supplemented with 10% FBS (Thermo Fisher Scientific), 100 U/ml penicillin and 100 μg/ml streptomycin (Invitrogen). HeLa Flp-In T-REx cells (a gift from Hemmo Meyer, University of Duisburg-Essen; referred to as HeLa cells throughout the article) were cultured in DMEM (Thermo Fisher Scientific, #31966021) supplemented with 10% FBS (Thermo Fisher Scientific), 100 U/ml penicillin and 100 μg/ml streptomycin (Thermo Fisher Scientific) (referred to as DMEM growth medium), containing 100 µg/ml zeocin (Invivogen) and 15 µg/ml blasticidin (Invivogen). HeLa Kyoto cells (a gift from Hemmo Meyer, University of Duisburg-Essen, Center of Medical Biotechnology, Essen, Germany) were grown in DMEM growth medium. Stable HeLa Flp-In T-REx cells expressing FAM129A^myc–APEX2^ under a doxycyline-inducible promotor were generated using the Flp-In T-REx system (Thermo Fisher Scientific) in combination with the pcDNA5/FRT/TO vector according to the manufacturer's protocol. DNA transfection was performed with transporter 5 (Kyfora Bio). Stable HeLa Flp-in T-REx FAM129A^myc–APEX2^ cells were selected and cultured in DMEM growth medium containing 250 µg/ml hygromycin B (Invivogen). After selection was completed, cells were maintained in DMEM growth medium containing 250 µg/ml hygromycin B and 15 µg/ml blasticidin (Invivogen). All cell lines were grown at 37°C and 5% CO_2_ and were regularly tested for mycoplasma infection using Venor GeM OneStep mycoplasma detection kit (Minerva Biolabs). All small-molecule stress treatments were performed using 1000× stocks of the molecules prepared in DMSO. For use, 11× stocks were prepared in the respective culture medium and added to seeded cells for the indicated time.

### Generation of ATF4 CRISPR/Cas9 KO cell line

CRISPR plasmids for *ATF4* KO were purchased from Santa Cruz Biotechnology [CREB-2 (ATF4) CRISPR/Cas9 KO Plasmid (h), CREB-2 (ATF4) HDR Plasmid (h)] and co-transfected into Ma-Mel-61a cells using Lipofectamine 3000 (Invitrogen, #L3000-008) according to the manufacturer's protocol. Briefly, 2×10^5^ cells were seeded into 6-well plates. After 24 h, cells were transfected with 1 µg of each plasmid complexed with 6 µl Lipofectamine 3000 in RPMI (Gibco). After 6 h, medium was replaced, and antibiotic selection was initiated with 1.5 µg/ml puromycin 24 h after transfection. Growing single-cell clones were isolated by manual picking and expanded under continued antibiotic selection. *ATF4* KO was confirmed by western blotting.

### Quantitative PCR

Total RNA was isolated from melanoma cells (<5×10^6^) using the RNeasy Plus Mini Kit with on-column DNase I treatment (Qiagen, #74134, #79254). cDNA was synthesized from 2 µg of RNA using the Verso cDNA Synthesis Kit (Thermo Fisher Scientific, #AB-1453/A). Quantitative PCR (qPCR) was performed in duplicates with 20 ng of template cDNA and 400 nM primers using the ABsolute qPCR SYBR Green Mix (Thermo Fisher Scientific, #AB-1158/B) on a Bio-Rad CFX96 Touch Real-Time PCR Detection System. *MLANA* expression was analyzed using Bio-Rad CFX Manager software and normalized to *RPL13A* expression. Primer sequences are shown in Table S6.

### Western blotting

Cells were harvested on ice in lysis buffer [50 mM Tris-HCl pH 7.5, 150 mM NaCl, 1 mM EDTA, 0.25% SDS, 1% NP-40, 0.5% Triton X-100, 1× protease inhibitor cocktail (Roche)]. Lysates were centrifuged for 10 min at 20,000 ***g*** at 4°C, the supernatant was collected and the protein concentration determined using the BCA Protein Assay Kit (Pierce). The supernatant was mixed with 4× SDS sample buffer (200 mM Tris-HCl pH 6.8, 8% SDS, 40% glycerol, 4% β-mercaptoethanol, Bromophenol Blue) and incubated at 95°C for 5 min. Protein extracts were applied to SDS–PAGE gels followed by transfer to a nitrocellulose membrane for 90 min at 75 V. Membranes were blocked in 5% non-fat dry milk in TBS containing 0.1% Tween-20 for 1 h prior to incubation with the primary antibody dilutions. Membranes were probed with primary antibodies diluted as listed in Table S3 in 3% BSA, 0.02% sodium azide, 0.1% TWEEN-20 in TBS followed by incubation with HRP-conjugated or fluorescently labeled secondary antibodies diluted in TBS, 0.1% Tween-20. Secondary antibodies were incubated for 45 min at room temperature (RT) before detection on a ChemiDoc MP imaging system (Bio-Rad) using ECL start (Cytiva) or ECL prime (Cytiva) western blotting detection reagents for HRP-conjugated antibodies. Band intensities were analyzed using Image Lab (Bio-Rad), and data were plotted in Prism (Graphpad).

### Collecting samples from cultured media

The growth medium of RPE1 cells was removed, and cells were washed twice with FBS-free medium before treatment. After treatment, the same volume of growth medium was collected for all samples and centrifuged at 1000 ***g*** for 5 min to precipitate possible cells or debris present in the growth medium. The same volume of supernatant for each sample was transferred to a new tube and mixed with 4× SDS sample buffer. Samples were incubated for 5 min at 95°C before SDS–PAGE and western blot analysis. SDS–PAGE gels were prepared with 0.5% 2,2,2-trichloroethanol (TCE) for visualization of proteins following electrophoresis. TCE signal was detected with the stain-free (UV light) settings of the ChemiDoc MP imaging system (Bio-Rad).

### Sample preparation for whole proteome analysis

HeLa Flp-in T-REx cells were grown in 6 cm dishes. Cells were treated with DMSO for 8 h and with nigericin for the last 1, 4 or 8 h. RPE1 cells were grown in 10 cm dishes and treated with DMSO or nigericin for 8 h. The cell layer was rinsed 2× with ice-cold DPBS (Gibco; 14190136). Cells were then scraped off in 1 ml ice-cold DPBS, transferred to 1.5 ml tubes and pelleted for 5 min at 1000 ***g***, at 4°C. The HeLa cell pellet was lysed in 50 mM HEPES pH 7.5, 150 mM NaCl, 1 mM EDTA, 0.5% SDS, 1% NP-40. The RPE1 cell pellet was lysed in 50 mM Tris-HCl pH 7.5, 150 mM NaCl, 1 mM EDTA, 0.25% SDS, 1% Triton X-100, 1× protease inhibitor cocktail (Roche). Cell debris was removed by centrifugation for 10 min at 10,000 ***g*** at 4°C, and the soluble fraction (protein extract) was subjected to sample preparation for MS analysis. Three to four replicates were analyzed for DMSO control and the 4 h nigericin treatment time point, and four replicates were analyzed for the 1 h and 8 h treatment time points.

After protein concentration measurement (Pierce BCA assay, 23225), 10 µg of the protein extracts were used for sample preparation for MS using the single-pot, solid-phase-enhanced sample-preparation (SP3) strategy (Hughes et al., 2019). All buffers and solutions were prepared with MS-grade water (Honywell). 10 µg of the protein extract was brought to a final volume of 37.5 µl with water and then supplemented with 4× SP3 lysis buffer [200 mM Hepes pH 8.0, 4% (w/v) SDS, 40 mM TCEP, 160 mM chloroacetamide]. The samples were then heated at 90°C for 6 min and cooled down to RT. In order to degrade nucleic acid, the samples were then incubated with 2 µl 3.5 U/µl Benzonase (#71206; Merck Millipore) in dilution buffer (20 mM Hepes pH 8.0, 2 mM MgCl_2_) at 37°C for 30 min with gentle agitation at 1500 rpm. After Benzonase treatment, 3 µl of a 50 µg/µl 1:1 mixture of hydrophilic (#45152105050250) and hydrophobic (#65152105050250) carboxylate-modified Sera-Mag SpeedBeads (Cytiva), which were washed twice with MS-grade water, were added to the samples and mixed by gently pipetting up and down. Protein binding to the beads was induced by the addition of an equal volume of pure ethanol (10 min, 1000 rpm, 24°C). After incubation, beads were collected by a brief centrifugation step (10 s, 200 ***g***), and the reaction vessels placed on a magnetic stand. Beads were allowed to migrate to the vessel wall for at least 5 min before the supernatant was removed. The beads were then taken up in 180 µl 80% ethanol and transferred to a fresh vessel. Subsequently, the beads were washed four times with 180 µl 80% (v/v) ethanol prior to the addition of 100 µl digestion enzyme mix [0.6 μg of trypsin (V5111; Promega) and 0.6 µg LysC (125-05061; FUJIFILM Wako Pure Chemical) in 25 mM ammonium bicarbonate]. Samples were incubated at 37°C for 19 h while shaking (1300 rpm). On the next day, the samples were briefly centrifuged (10 s, 200 ***g***) and placed on a magnet for 5 min. The clear solution containing the tryptic peptides was transferred to a new reaction vessel. The beads were taken up in 47 µl 25 mM ammonium bicarbonate and incubated while shaking (2 min, 1000 rpm). The reaction vessels were then placed on a magnetic stand again, and after 5 min the cleared supernatant was collected and combined with the recovered first peptide mix, followed by the addition of formic acid (FA) to a final concentration of 2% (v/v) for trypsin inactivation.

### FAM129A^myc–APEX2^ proximity biotinylation and streptavidin pull-down

For APEX2 proximity biotinylation, HeLa Flp-In T-REx FAM129A^myc–APEX2^ cells were cultured in 10 cm dishes (four DMSO samples, four nigericin samples per experiment). FAM129A^myc–APEX2^ expression was induced with 100 ng/ml doxycycline 18 h prior to treatment with 1 µM nigericin or DMSO for an additional 4 h. In the last 30 min of treatment, 500 µM biotin phenol (Sigma-Aldrich, #SML2135) was added. Culture dishes were removed from the incubator and equilibrated to RT for 5 min. Then, 1 mM H_2_O_2_ was added and incubated for exactly 1 min before the solution was replaced by quenching buffer (10 mM sodium ascorbate, 10 mM sodium azide, 5 mM Trolox in PBS). Samples were washed two times with quenching buffer and incubated for 15 min on ice in the last washing step. Quenching buffer was removed, and the cell layer was washed two times with PBS. Cells were taken off the dishes by scraping in 1 ml PBS, transferred to reaction tubes and sedimented at 400 ***g*** for 3 min. Cells were lysed in 50 mM Tris-HCl pH 7.5, 150 mM NaCl, 0.1% SDS, 0.5% sodium deoxycholate, 1% Triton X-100, 10 mM sodium azide, 10 mM sodium ascorbate, 5 mM Trolox, 1× protease inhibitor cocktail (Roche). Cell debris was removed by centrifugation for 10 min at 15,000 ***g*** at 4°C. Protein extracts were incubated with magnetic streptavidin beads (Thermo Fisher Scientific) for 1 h at RT. Beads were then washed twice with RIPA buffer [50 mM Tris-HCl pH 7.5, 150 mM NaCl, 0.1% SDS, 0.5% sodium deoxycholate, 1% Triton X-100, 1× protease inhibitor cocktail (Roche)], followed by single washes with 1 mM KCl, with 0.1 M Na_2_CO_3_, with urea buffer (2 mM urea, 10 mM Tris-HCl pH 8.0) and additional two washes with RIPA buffer. In the last washing step, the samples were transferred to fresh reaction tubes. Biotinylated proteins were eluted from the beads by incubation in 200 mM HEPES pH 8.0, 4% SDS, 40 mM TCEP, 160 mM chloroacetamide, 2 mM biotin for 5 min at 95°C. Eluates were flash-frozen in liquid nitrogen and stored at −70°C until processing for MS.

Pull-down samples were prepared following the SP3 strategy (Hughes et al., 2019). The samples were washed on beads and then taken up in a slightly modified SP3 Lysis buffer (final concentrations 1% SDS, 10 mM TCEP, 40 mM chloroacetamide, 50 mM HEPES, 2 mM biotin; final volume 100 µl). Samples were heated at 90°C for 5 min and cleared by centrifugation. Supernatant was transferred to a fresh reaction vessel. Then, 3 µl of a 50 µg/µl 1:1 mixture of hydrophilic (#45152105050250) and hydrophobic (#65152105050250) carboxylate modified Sera-Mag SpeedBeads (Cytiva), which were washed twice with MS-grade water, were added to the samples. Afterwards, the samples were mixed shortly (1 min, 1000 rpm) and collected by short centrifugation (10 s, 200 ***g***). Protein binding was induced by the addition of an equal volume of pure ethanol (10 min, 1000 rpm), the beads were collected by a brief centrifugation step (10 s, 200 ***g***), and the reaction vessels placed on a magnetic stand. Beads were allowed to migrate to the vessel wall for at least 5 min before the supernatant was removed. The beads were then taken up in 180 µl 80% ethanol and transferred to a fresh reaction vessel. Subsequently the beads were washed four times with 180 µl 80% (v/v) ethanol prior to the addition of 100 µl digestion enzyme mix [0.6 μg of trypsin (V5111; Promega) and 0.6 µg LysC (125-05061; FUJIFILM Wako Pure Chemical) in 25 mM ammonium bicarbonate]. Samples were incubated at 37°C for 19 h while shaking (1300 rpm). On the next day, the samples were briefly centrifuged (10 s, 200 ***g***) and placed on a magnet for 5 min. The clear solution containing the tryptic peptides was transferred to a fresh reaction vessel. The beads were taken up in 47 µl 25 mM ammonium bicarbonate and incubated while shaking (2 min, 1000 rpm). The vessels were then placed on a magnetic stand, and after 5 min the cleared supernatant was collected and combined with the recovered first peptide mix, followed by the addition of FA to a final concentration of 2% (v/v) for trypsin inactivation.

#### Sample clean-up for liquid chromatography–tandem MS

In preparation for liquid chromatography–tandem MS (LC–MS/MS), all digests were desalted on home-made C18 StageTips containing two layers of an octadecyl silica membrane (CDS Analytical) (Rappsilber et al., 2007). All centrifugation steps were carried out at RT. The StageTips were first activated and equilibrated by passing 50 μl methanol (600 ***g***, 2 min), 80% (v/v) acetonitrile (ACN) with 0.5% (v/v) FA (600 ***g***, 2 min), and 0.5% (v/v) FA (600 ***g***, 2 min) over the tips. Next, the acidified tryptic digests were passed over the tips (800 ***g***, 3 min). The immobilized peptides were then washed with 50 μl and 25 μl 0.5% (v/v) FA (800 ***g***, 3 min). Bound peptides were eluted from the StageTips by application of two rounds of 25 μl 80% (v/v) ACN with 0.5% (v/v) FA (800 ***g***, 2 min). Peptide samples were then dried using a vacuum concentrator (Eppendorf) and the peptides were dissolved in 15 μl 0.1% (v/v) FA prior to analysis by MS.

#### LC–MS/MS settings

MS experiments were performed on an Orbitrap LUMOS instrument (Thermo) coupled to an EASY-nLC 1200 or Vanquish Neo ultra-performance liquid chromatography (UPLC) system (Thermo). The UPLC was operated in the one-column mode. The analytical column was a fused silica capillary [75 µm×46 cm for full proteome analysis-mass spectrometry (FPA-MS) of HeLa cells, or 75 µm×28 cm for FPA-MS of RPE1 cells and proximity-dependent biotinylation MS (PDB-MS)] with an integrated fritted emitter (New Objectives PF360-75-15-N-5 or CoAnn ICT36007515F-50-5) packed in-house with 1.9 µm Reprosil-Pur 120 C18-AQ (Dr. Maisch; for FPA-MS of HeLa) or 1.7 µm Kinetex C18-XB core shell beads (Phenomenex; for FPA-MS of RPE1 and PDB-MS). The analytical column was encased by a column oven (Sonation PRSO-V2) and attached to a nanospray flex ion source (Thermo). The column oven temperature was set to 50°C during sample loading and data acquisition. The LC system was equipped with two mobile phases. All solvents were of UPLC grade (Honeywell). Peptides were directly loaded onto the analytical column with a maximum flow rate that would not exceed the set pressure limit of 980 bar (usually around 0.4–0.6 µl/min). Peptides were subsequently separated on the analytical column by running a gradient of solvent A and solvent B. Gradient length and composition, mobile phase composition and LC–MS settings are available upon request.

### Peptide and protein identification using MaxQuant

RAW spectra were submitted to an Andromeda (Cox et al., 2011) search in MaxQuant (v1.6.10.43 or v2.0.3.0) using the default settings (Cox and Mann, 2008). Label-free quantification and match-between-runs was activated (Cox et al., 2014). The MS/MS spectra data were searched against the *Homo sapiens* reference databases UP000005640_9606.fasta (one protein per gene 20621 entries, downloaded October 2020) or UP000005640_2-HomoSapiens(08-2022).fasta (79759 entries, downloaded August 2022). For proximity-dependent biotinylation experiments we also included the dedicated database ACE_0798_SOI_v01.fasta containing only the APEX2 sequence. All searches included a contaminants database search (as implemented in MaxQuant, 245 entries). The contaminants database contains known MS contaminants and was included to estimate the level of contamination. Andromeda searches allowed oxidation of methionine residues (16 Da) and acetylation of the protein N terminus (42 Da) as dynamic modifications, and the static modification of cysteine (57 Da, alkylation with iodoacetamide). Enzyme specificity was set to ‘Trypsin/P’ with two missed cleavages allowed. The instrument type in Andromeda searches was set to Orbitrap, and the precursor mass tolerance was set to ±20 ppm (first search) and ±4.5 ppm (main search). The MS/MS match tolerance was set to ±0.5 Da. The peptide spectrum match FDR and the protein FDR were set to 0.01 (based on a target–decoy approach). Minimum peptide length was seven amino acids. For protein quantification, unique and razor peptides were allowed. Modified peptides were allowed for quantification. The minimum score for modified peptides was 40. Label-free protein quantification was switched on, and unique and razor peptides were considered for quantification with a minimum ratio count of 2. Retention times were recalibrated based on the built-in nonlinear time-rescaling algorithm. MS/MS identifications were transferred between LC–MS/MS runs with the ‘match between runs’ option, in which the maximal match time window was set to 0.7 min and the alignment time window set to 20 min. The quantification is based on the ‘value at maximum’ of the extracted ion current. At least two quantitation events were required for a quantifiable protein.

### Proteomics data analysis

Further analysis and filtering of the results was performed in Perseus v1.6.10.0. (Tyanova et al., 2016). Comparison of protein group quantities (relative quantification) between different MS runs is based solely on the label-free quantifications (LFQs) as calculated by the MaxQuant MaxLFQ algorithm (Cox et al., 2014). The FAM129A biotinylation experiment was performed twice. For the quantification of each experiment, we combined related biological replicates to categorical groups and investigated only those proteins that were found in at least one categorical group in a minimum of four out of four biological replicates. From these datasets, we then further excluded common contaminants typically identified in streptavidin pull-down studies (Mellacheruvu et al., 2013) by removing all proteins that were identified as contaminants in eight of eight comparable experiments (experiments CC851–CC859 of the CRAPome database). Significant hits (FDR≤0.01 and s0 of 0.1) that were identified in both experiments were used in the final analysis (228 proteins with decreased abundance, 68 proteins with increased abundance). Fig. 3B shows the results of dataset 2 with the filtered proteins highlighted in color. Fig. 3C shows 26 of 68 hits with increased proximity to FAM129A^myc–APEX2^ upon nigericin treatment that localize to the ER, Golgi or endosomes according to the respective GO term cellular component. For the analysis of the whole proteome samples upon nigericin treatment, we investigated only those proteins that were found in at least one categorical group in a minimum of three biological replicates within one group. In Fig. 1F, the Log2 FC of the 17 Golgi-associated hits from Table S1, sheet1 (HeLa, 8 h nigericin) were plotted against the Log2 FC of the same proteins in Table S1, sheet2 (RPE1, 8 h nigericin), and data extracted from the publicy available MassIVE dataset MSV000091726 (doi:10.25345/C5HT2GN5X; oxybipin-2; He et al., 2025) and PXD040694 deposited in the PRIDE repository (orpinolide; Cigler et al., 2025).

### Fluorescence microscopy of fixed cells

For microscopy experiments, cells were grown on fibronectin-coated coverslips (Merck, #F0895) and, after the indicated treatment, fixed in 4% paraformaldehyde in PBS. Cells were permeabilized and then blocked in 10% FBS in PBS containing 0.01% Triton X-100 for 45 min at RT, followed by incubation with primary antibodies (see Table S4). After incubation with secondary antibodies for 45 min at RT, nuclei were stained with 5 µg/ml Hoechst 33342 in PBS for 45 min at RT. Coverslips were mounted on microscopy slides in Vectashield mounting medium (Vector Laboratories). Images were acquired at a Leica TCS SP8 HCS A and Leica TCS SP8X Falcon using HC PL APO CS2 objectives with 63× magnification and 1.4 numerical aperture. Images were processed using OMERO (Allan et al., 2012).

### Preparation of cells for live-cell imaging

For live-cell imaging studies, HeLa Kyoto cells were electroporated with plasmid DNA using the NEPA21 electroporator (Nepa Gene). For each electroporation, cells were washed twice with Opti-MEM (Gibco) then resuspended in Opti-MEM, and 1×10^6^ cells were mixed with 5.5 µg ST6GAL1–mCherry plasmid DNA and 11 µg Str-Ii_IRES_VSVG-SBP-EGFP plasmid DNA in a 2 mm electroporation cuvette. The two poring pulses were 150 V, 2.5 ms length, 50 ms pulse interval, with decay rate of 10%. The five transfer pulses were 20 V, 50 ms length, 50 ms interval, with a decay rate of 40%. For RUSH assays of Str-Ii_IRES_VSVG-SBP-EGFP, GRASP65–SNAP and FAM129A–mRFP–FLAG, cells were electroporated as described above (5.5 µg plasmid DNA for GRASP65–SNAP and FAM129A–mRFP–FLAG each). GRASP65–SNAP was labeled with 2 µM SNAP-Cell 647 SiR (NEB, #S9102S) 30 min prior to the microscopy experiment, according to the manufacturer's instructions. For live-cell imaging of FAM129A–mRFP–FLAG and TGN46–GFP, HeLa Kyoto cells were electroporated with 5.5 µg plasmid DNA each. Electroporated cells were grown in µ-slide 4-well dishes (Ibidi, #80426) for 24 h. For RUSH assays, cells were treated with 1 µM nigericin for 4 h or left untreated prior to time-lapse confocal imaging.

### Time-lapse spinning-disk confocal imaging

Live-cell imaging was performed on an inverted Nikon Ti-E microscope (Nikon Europe B.V., Amstelveen, the Netherlands) equipped with a Yokogawa CSU-X1 spinning disk confocal unit (Yokogawa Electric Corporation, Tokyo, Japan) and controlled by Andor iQ software (version 3.7.0; Andor Technology, Belfast, UK). Images were acquired using an Andor Zyla 4.2 sCMOS camera (Andor Technology, Belfast, UK) at 1024×1024 pixels (16-bit, 1×1 binning; pixel size ∼0.095 µm) with a Nikon CFI Apo TIRF 60×/1.49 NA oil immersion objective (Nikon Europe B.V., Amstelveen, the Netherlands). Single *z*-planes were acquired throughout. Cells were maintained at 37°C and 5% CO_2_ in an EMBLEM microscope incubator box (EMBLEM, Heidelberg, Germany). Imaging positions were defined prior to treatment. For RUSH assays, biotin (Sigma-Aldrich, #B4639) was added as a 2× stock (final concentration 40 µM) on stage immediately before acquisition. The 1 µM nigericin treatment was maintained for the duration of the acquisition. For colocalization studies, a 2× stock of nigericin (final concentration 1 µM), or DMSO as a control, was added directly on stage immediately before acquisition.

Excitation was provided by an Andor ILE multimode laser engine (50 mW 488 nm and 561 nm lasers, 140 mW 637 laser; Andor Technology, Belfast, UK). Emission was detected using Semrock BrightLine filters FF01-512/630-25 for 488 nm, FF01-593/40-25 for 561 nm and FF02-685/40-25 for 637 nm excitation (Semrock, Rochester, NY, USA). Imaging was performed with the following settings: VSVG–SBP–EGFP (488 nm, 20%, 160 ms exposure time), TGN46–GFP (488 nm, 12%, 100 ms exposure time), FAM129A–RFP (561 nm, 20%, 160 ms exposure time) and GRASP65–SNAP–SiR-647 (637 nm, 5%, 50 ms exposure time). Time-lapse recordings were acquired at 1 min intervals for 90 min for RUSH assays and at 2 min intervals for 240 min for colocalization assays. Between 13 and 17 (RUSH assay), or 30 to 32 (colocalization study), *XY* fields were recorded per experiment, depending on cell density and spatial distribution, with hardware autofocus (Perfect Focus System; Nikon Europe B.V.) active during acquisition. Two-frame averaging was applied where indicated.

### Image analysis and RUSH assay quantification

Time-lapse fluorescence images were analyzed using CellProfiler (version 4.2.5) (Stirling et al., 2021) Single cells were segmented using the integrated Cellpose algorithm (RunCellpose plugin, Cellpose version 2, cyto2 model; Stringer et al., 2021) and tracked over time to generate unique cell track identifiers. For RUSH assays, the Golgi regions (as defined by ST6Gal1–mCherry or GRASP65–SNAP, depending on the experiment) were identified using intensity-based thresholding of the Golgi marker channel. Detected Golgi objects were consolidated to obtain a unified Golgi compartment per cell prior to quantification. A binary Golgi mask was applied to the VSVG–SBP–EGFP channel to generate a Golgi-restricted VSVG–SBP–EGFP image. The mean fluorescence intensity was measured per cell for both the Golgi-masked VSVG–SBP–EGFP image and the total cellular VSVG–SBP–EGFP signal. The Golgi localization ratio was calculated as the mean VSVG–SBP–EGFP intensity within the Golgi mask divided by the mean total cellular VSVG–SBP–EGFP intensity for each cell and time point. To restrict analysis to cells within a comparable expression range – and to exclude untransfected cells, background signal, or potential saturation or overexpression artifacts – only cells with a mean total VSVG–SBP–EGFP intensity at t_0_ between≥0.0018 and <0.0035 arbitrary units (a.u.) were included. For each individual cell track, baseline values at t_0_ were subtracted from all subsequent time points. Data were subsequently normalized such that t_0_ was defined as 0% and the maximum value within each track was set to 100%. For colocalization experiments with TGN46^GFP^ and FAM129A^RFP^, the Pearson correlation coefficient (PCC) was calculated for each segmented cell and time point in CellProfiler. To detect true fluorescence signals and avoid background-driven correlations, only cells with a mean fluorescence intensity of at least 0.0018 a.u. in both channels at t_0_ were included. Only cells that were successfully tracked throughout the imaging sequence were retained for analysis. All downstream data processing and graphical representations were performed using GraphPad Prism version 10.6.1.

### Bioinformatic analysis of *FAM129A* expression in RNA sequencing datasets and in single-cell RNA sequencing data

Analyses of previously published melanoma transcriptomic datasets were performed using the web tool available at https://tools.hornlab.org/cru337phenotime/ (Philip et al., 2023). Data from Tsoi et al. (NCBI GEO accession GSE80829, FPKM format; Tsoi et al., 2018) and Wouters et al. (GSE134432, normalized counts; Wouters et al., 2020) were used. Cell lines were classified as *MITF*-high or *MITF*-low, and *FAM129A* expression was compared between groups using a two-sided Wilcoxon rank-sum test. Genes significantly correlated with *FAM129A* (FDR<0.05) were identified by Spearman correlation analysis in the Tsoi et al. dataset. GO enrichment analysis was performed using the online tool Enrichr (Chen et al., 2013; Kuleshov et al., 2016; Xie et al., 2021) on the top 100 positively correlated genes. Analyses of previously published melanoma single-cell RNA sequencing datasets (Pozniak et al., 2024; https://doi.org/10.48804/GSAXBN) were performed using the webtool at https://marine-lab.shinyapps.io/Human_melanoma_scRNASeq/. Graphs were generated using R version 4.4.1.

## Supplementary Material



10.1242/joces.264535_sup1 Supplementary information

Table S1. Proteome changes upon nigericin treatment

Table S2. Proteomics data proximity biotinylation of FAM129A-myc-APEX2
