# Supplementary material for: Stress-induced proteome remodeling at the Golgi–endosome interface
Source: J Cell Sci. 2026 May 11;139(9):jcs264535. doi: 10.1242/jcs.264535 (PMC13245918; doi:10.1242/jcs.264535)
Supplement: Supplementary information [file joces-139-264535-s1.pdf]

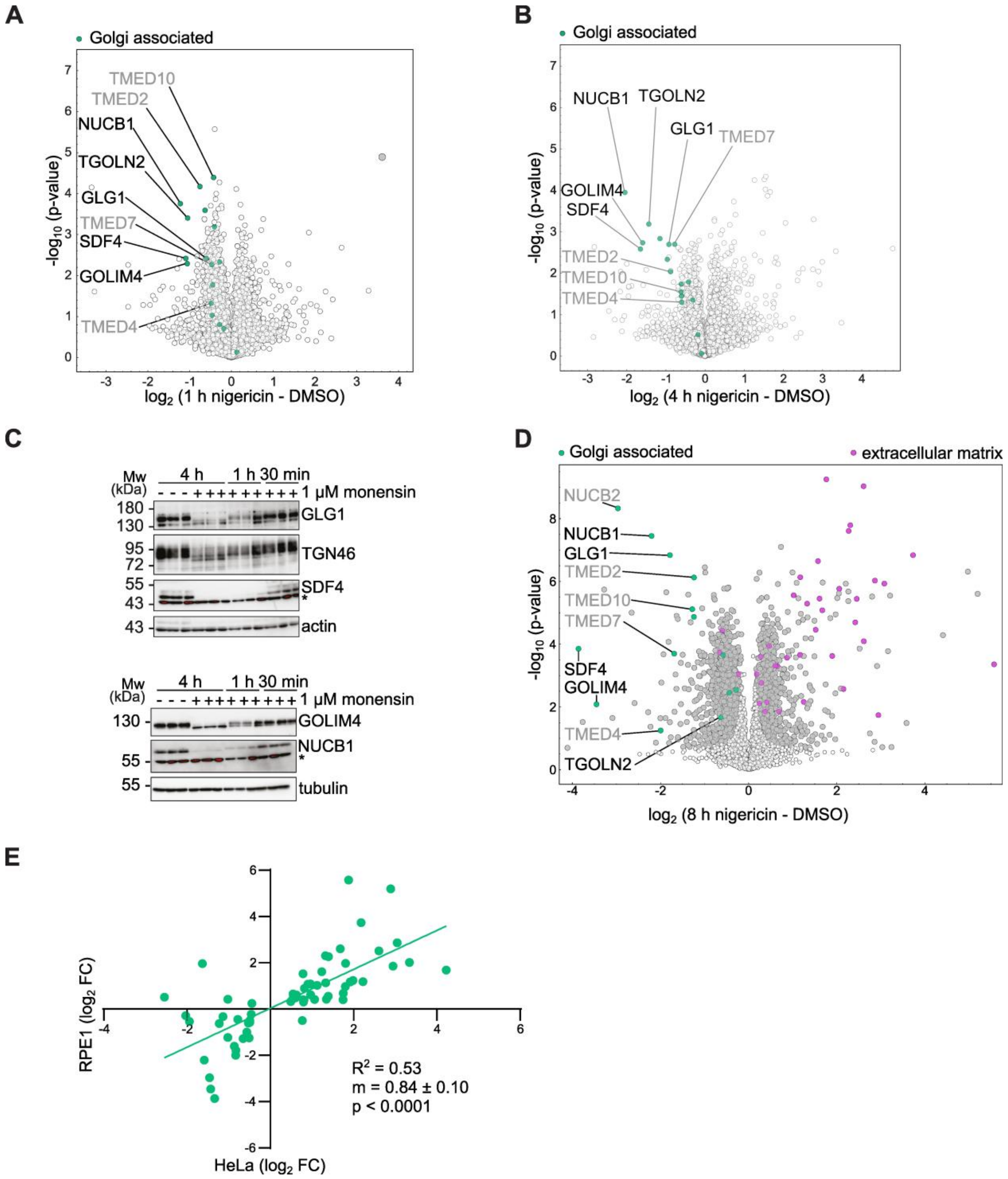

Fig. S1. Proteomic analysis of ionophore-induced Golgi stress. (**A-B**) Volcano plots representing quantitative MS data obtained from HeLa Flp-in T-REx cells treated with 1 µM nigericin for (A) 1 h or (B) 4 h vs DMSO control. Significant hits are highlighted in dark grey (FDR=0.05, S0=0.1), Golgi proteins decreased upon nigericin treatment are highlighted in green. (**C**) Western blot of RPE1 cells upon 1 µM monensin treatment. (**D**) Volcano plot representing quantitative MS data obtained from RPE1 cells treated with 1 µM nigericin for 8 h. Significant hits are highlighted in dark grey (FDR=0.05, S0=0.1), Golgi proteins decreased upon nigericin treatment are highlighted in green. Proteins that localize to the extracellular matrix are shown in magenta. (**E**) Correlation plot of proteins with significant changes in abundance upon treatment with 1 µM nigericin for 8 h in RPE1 and HeLa cells.

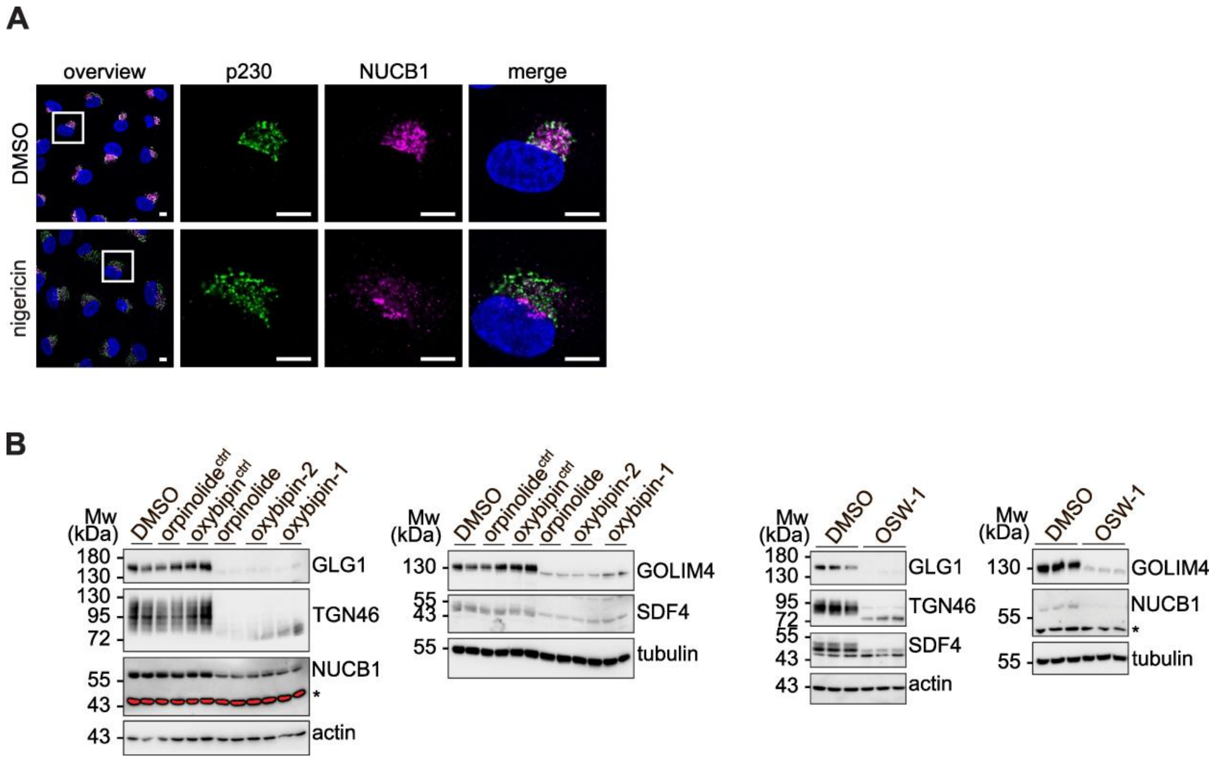

Fig. S2. Small molecule-induced Golgi stress in RPE1 cells. (**A**) Representative confocal sections depicting immunofluorescence of NUCB1 (magenta) and the Golgi marker p230 (green) in RPE1 cells upon 1 µM nigericin treatment for 30 min. Nuclei were stained with Hoechst (blue). Scale bars=10 µm. (**B**) Western blot analysis of RPE1 cells upon 1 µM treatment of the indicated compounds for 18 h.

Journal of Cell Science • Supplementary information

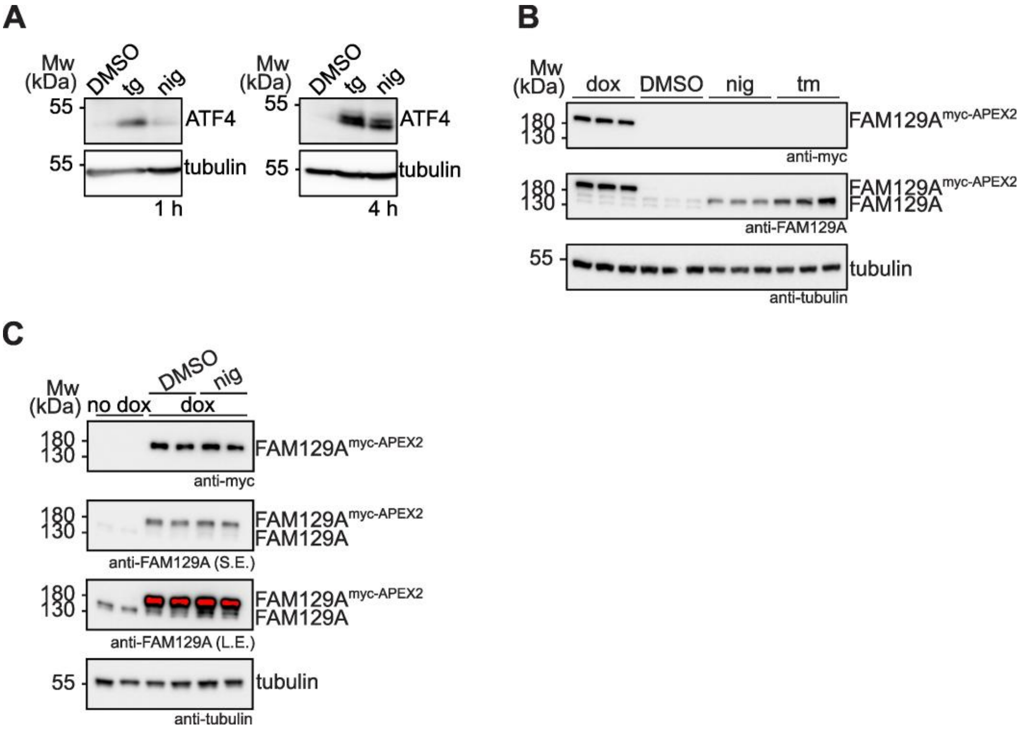

Fig. S3. FAM129A and nigericin-induced stress in HeLa cells. (**A**) Western blot of HeLa cells treated with 1 µM nigericin (nig), 0.5 µM thapsigargin (tg) or DMSO for the indicated times. (**B**) Western blot of HeLa Flp-In T-REx FAM129A$^{myc\text{-}APEX2}$ cells treated with 100 ng/ml doxycycline (dox), 1 µM nigericin (nig), 5 µg/ml tunicamycin (tm) or DMSO for 18 h. (**C**) Western blot of HeLa Flp-In T-REx FAM129A$^{myc\text{-}APEX2}$ cells treated with 100 ng/ml doxycycline for 22 h and 1 µM nigericin or DMSO for the last 4 h.

Journal of Cell Science • Supplementary information

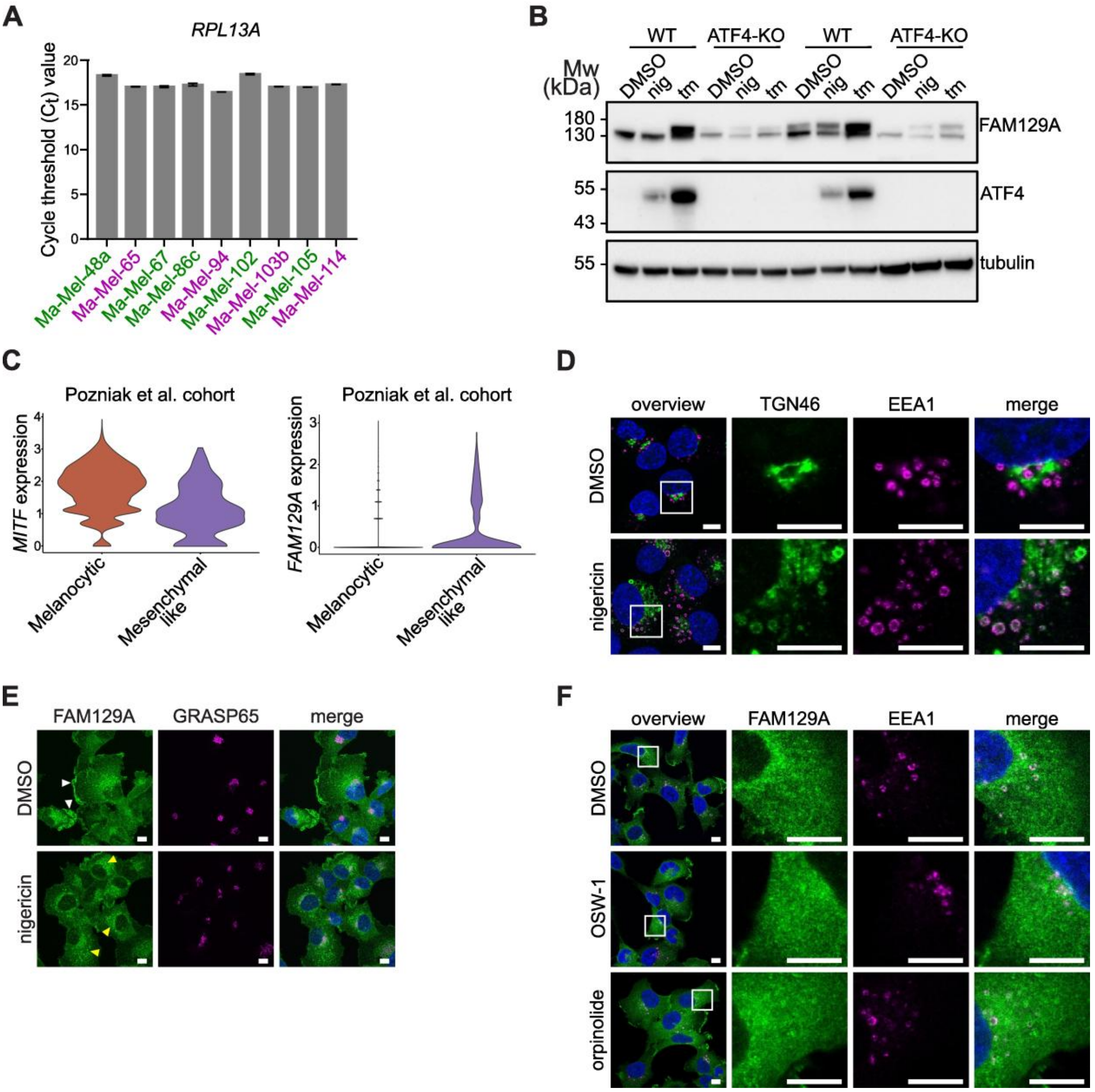

Fig. S4. FAM129A levels and expression pattern in melanoma cells. (**A**) Control qPCR related to Figure 4A. Analysis of *RPL13A* expression in the different patient-derived melanoma cells (magenta – dedifferentiated cell lines, green – differentiated cell lines) shows comparable *RPL13A* expression in all cell lines. Graphs represent the mean ± S.E.M. (**B**) Western blot analysis of Ma-Mel-61a WT and ATF4 KO cells treated with 1 µM nigericin (nig) or 5 µg/ml tunicamycin (tm) for 18 h. Replicates are from two independent experiments. (**C**) Violin plot showing *MITF* (a differentiation marker) and *FAM129A* expression in differentiated (melanocytic) and mesenchymal-like (dedifferentiated) melanoma cells from the Pozniak et al. cohort. (**D-F**) Representative confocal sections depicting immunofluorescence of Ma-Mel-94 cells treated with 1 µM nigericin. (D) TGN46 (green) and EEA1 (magenta), treated for 1 h. (E) FAM129A (green) and the Golgi marker GRASP65 (magenta), treated for 4 h. White and yellow arrows indicate enrichment of FAM129A at the plasma membrane or endomembranes, respectively. (F) FAM129A (green) or EEA1 (magenta) treated with 1 µM OSW-1, 1 µM orpinolide or DMSO for 6 h. Nuclei were stained with Hoechst (blue). Scale bars=10 µm.

Journal of Cell Science • Supplementary information

Table S1. Proteome changes upon nigericin treatment

Available for download at
https://journals.biologists.com/jcs/article-lookup/doi/10.1242/jcs.264535#supplementary-data

Table S2. Proteomics data proximity biotinylation of FAM129A-myc-APEX2

Available for download at
https://journals.biologists.com/jcs/article-lookup/doi/10.1242/jcs.264535#supplementary-data

Table S3. Antibodies used for western blot

antibody	dilution	Supplier and Catalogue number	RRID
anti-GLG1	1:500	#HPA010815	AB_2232486
anti-GOLIM4	1:500	#HPA001677	AB_1079017
anti-TGN46	1:4000	Proteintech (#66477-1-Ig)	AB_2881843
anti-NUCB1	1:1000	#HPA008176	AB_1845923
anti-SDF4	1:1000	Invitrogen (#PA5-120363)	AB_2913935
anti-actin	1:1000	Santa Cruz Biotechnology (#sc-47778)	AB_626632
anti-tubulin	1:1000	Sigma-Aldrich (#T9026)	AB_477593
anti-FAM129A	1:500	Signalway Antibody (#21401)	AB_895743
anti-ATF4	1:1000	Cell signaling (#11815)	AB_2616025
Goat anti-mouse HRP	1:20.000	Thermo Scientific (#31444)	AB_228321
Goat anti-rabbit HRP	1:20.000	Thermo Scientific (#31460)	AB_228341
Goat anti-mouse (Alexa488)	1:10.000	Thermo Fisher Scientific (#A-11001)	AB_2534069

Journal of Cell Science • Supplementary information

Table S4. Antibodies used for Immunofluorescence

Antibody	Dilution	Supplier and Catalogue number	Incubation
anti-TGN46	1:100	Proteintech (#66477-1-Ig)	1 h at RT
anti-NUCB1	1:500	#HPA008176	o/n at 4 °C
anti-SDF4	1:200	Proteintech (#10517-1-AP)	o/n at 4 °C
anti-EEA1	1:500	BD Biosciences (#610457, RRID:AB_397830)	1 h at RT
anti-GRASP65	1:250	Santa Cruz Biotechnology (#sc-374423, RRID:AB_10991322)	1 h at RT
anti-p230	1:100	BD Biosciences (#611281, RRID:AB_398809)	1 h at RT
anti-FAM129A	1:200	#HPA028657, RRID:AB_10601680	1 h at RT
Goat anti-mouse (Alexa488)	1:500	Thermo Fisher Scientific (#A-11001, RRID:AB_2534069)	45 min at RT
Goat anti-mouse (Alexa 546)	1:500	Thermo Fisher Scientific (#A-11003, RRID:AB_2534071)	45 min at RT
Goat anti-rabbit (Alexa488)	1:500	Thermo Fisher Scientific (#A-11008, RRID:AB_143165)	45 min at RT
Goat anti-rabbit (Alexa 546)	1:500	Thermo Fisher Scientific (#A-11010, RRID:AB_2534077)	45 min at RT
Goat anti-rabbit (Alexa 633)	1:500	Thermo Fisher Scientific (#A-21070, RRID:AB_2535731)	45 min at RT

Table S5. Primers used for generation of DNA constructs

Primer	Sequence (5’-3’)
FAM129A into pcDNA3 HindIII site (fw)	GGGAGACCCAAGCTTGGATCCGCCACCATGGGCGGCTCAGCCT
FAM129A with GSSG linker overlap with myc-APEX2 XhoI site (rev)	TTCTTCAGAAATAAGTTTTTGTTCCATTCCACTGCTGCCGCTAGC
FAM129A-myc-APEX2 into pcDNA5/FRT/TO HindIII site (fw)	GGACTCTAGCGTTTAAACTTAAGCTTGCCACCATGGGCGGCTCA GCCTC
FAM129A-myc-APEX2 into pcDNA5/FRT/TO NotI site (rev)	TAGACTCGAGCGGCCGCCTAGGCATCAGCAAACCCAAGCT
GRASP65 into pcDNA3 HindIII site (fw)	ATAGGGAGACCCAAGCTTGCCACCATGGGCCTGGGCGTCA
GRASP65 overlap with SNAP-tag n-terminus (rev)	TTTCGCAGTCTTTGTCCATGCCTCCTTCTGTGGTAGAGATCTGGG CC
SNAP-tag (fw)	ATGGACAAAGACTGCGAAATGAAGC
SNAP-tag into pcDNA3 XhoI site (rev)	TCTAGATGCATGCTCGAGTCACCCAGGCTTGCCCAGTC

Journal of Cell Science • Supplementary information

Table S6. Primers used for qPCR

Primer	Sequence (5’-3’)
RPL13A fw	GGTGGTCGTACGCTGTG
RPL13A rev	GGTCCGCCAGAAGATGC
MLANA fw	CCAATGCTCCACCTGCTTAT
MLANA rev	AGGTGTCTCGCTGGCTCTTA

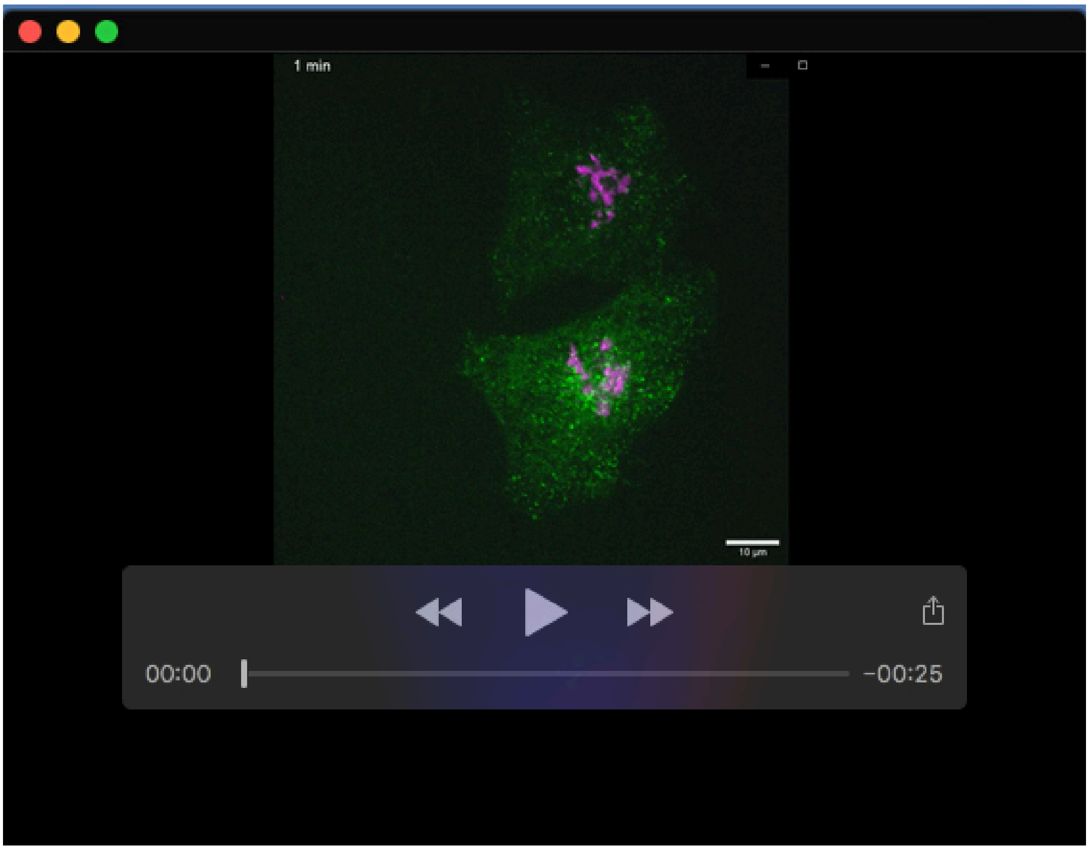

Movie 1. HeLa Kyoto cells expressing Str-li_IRES_VSVG-SBP-EGFP (green) and St6Gal1-mCherry (magenta). Cells were left untreated prior to biotin addition and monitored by time-lapse spinning disk confocal imaging. One picture was taken every minute.

Journal of Cell Science • Supplementary information

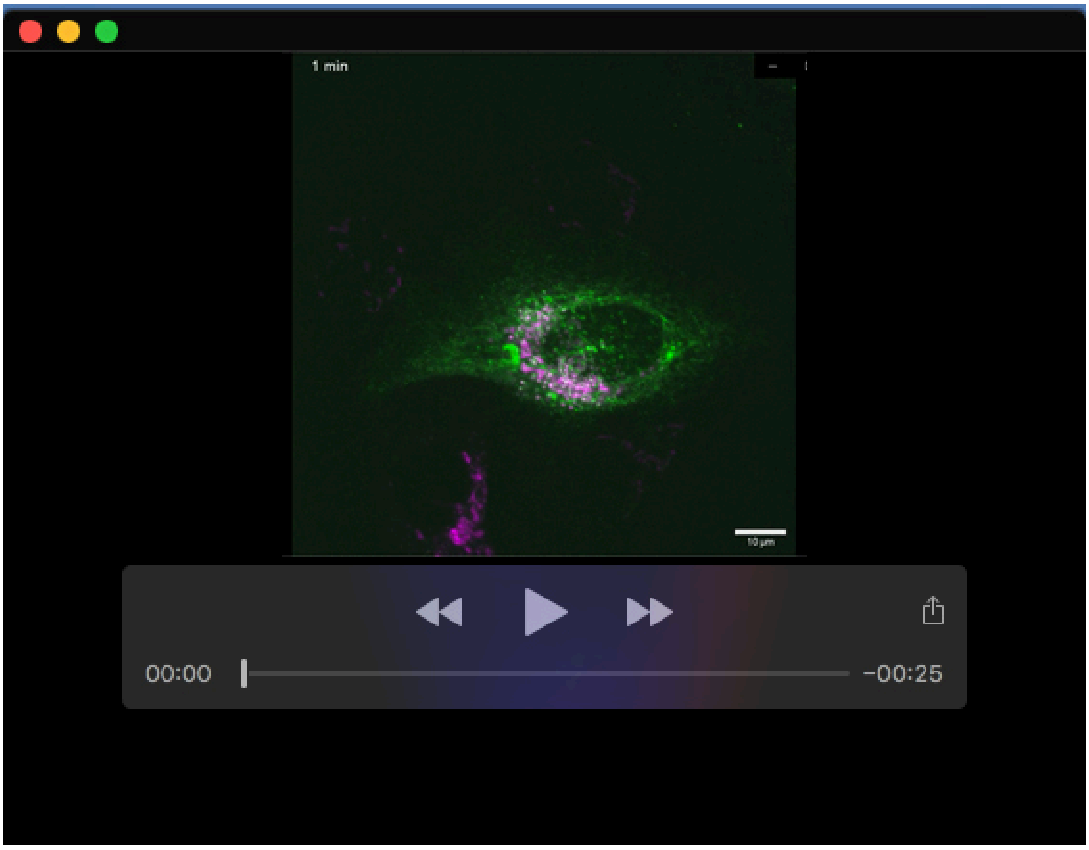

Movie 2. HeLa Kyoto cells expressing Str-li_IRES_VSVG-SBP-EGFP (green) and St6Gal1-mCherry (magenta). Nigericin treatment (1 µM) started 4 h prior to biotin addition and cells were monitored by time-lapse spinning disk confocal imaging. One picture was taken every minute.

Journal of Cell Science • Supplementary information
