# Supplementary material for: Stress-induced proteome remodeling at the Golgi–endosome interface
Source: J Cell Sci. 2026 May 11;139(9):jcs264535. doi: 10.1242/jcs.264535 (PMC13245918; doi:10.1242/jcs.264535)
Supplement: Supplementary file 1 [file joces-139-264535_review_history.pdf]

Journal of
Cell Science

Stress-induced proteome remodeling at the Golgi-endosome interface

Gina Simon, Savina Abraham-Pol, Melisa Dendusic, Nina Schulze, Simone Stupia, Michelle Koci, Lana Buzuk, Beatrice Thier, Philine Steinbach, Hai Trinh, Jasmin Schillinger, Sabrina Ninck, Farnusch Kaschani, Markus Kaiser, Michael Ehrmann, Annette Paschen and Doris Hellerschmied
DOI: 10.1242/jcs.264535

Editor: Robert Parton

Review timeline

Original submission:	28 October 2025
Editorial decision:	1 December 2025
First revision received:	2 March 2026
Accepted:	5 April 2026

Original submission

First decision letter

MS ID#: jcs.264535

MS TITLE: Stress-induced proteome remodeling at the Golgi-endosome interface

AUTHORS: Doris Hellerschmied; Gina Simon; Savina Abraham-Pol; Simone Stupia; Michelle Koci; Hai Trinh; Jasmin Schillinger; Sabrina Ninck; Farnusch Kaschani; Markus Kaiser; Michael Ehrmann; Annette Paschen

ARTICLE TYPE: Short Report

Dear Dr Hellerschmied,

We have now reached a decision on the above manuscript.

As you will see, the reviewers raised a number of concerns but both felt that a revised manuscript could be suitable for publication as a Short Report. We have completed the consultation session between the reviewers, which allows them to see the concerns and proposed revisions, and we all feel that all the suggested revisions are required and are feasible in a reasonable timeframe. I hope that you can deal satisfactorily with the criticisms on revision and I would be pleased to see a revised manuscript. We would then return it to the reviewers.

Reviewer 1

Advance summary and potential significance to field

In this manuscript submitted as a short report to the Journal of Cell Science and titled « Stress-induced proteome remodeling at the Golgi-endosome interface », Simon and colleagues describe the impact of various stress conditions on the remodeling of the proteome at the interface between the Golgi complex and endosomes.

© 2026. Published by The Company of Biologists under the terms of the Creative Commons Attribution License (https://creativecommons.org/licenses/by/4.0/).

The manuscript is well written and experimentally sound. Data are of great interest and value for the community. The authors document the molecular events occuring upon stress in the Golgi apparatus and for that use a proteomics approach combined with the use of select stress inducing drugs. One can regret that the functional impact of such stress response (through protein relocalization) on protein secretion is not interrogated, especially regarding the central dogma of the manuscript which is that stress (and Golgi stress) induces the remodeling of the secretory pathway.

Comments
* Most if not all endosomal stainings are performed using anti EEA1 antibodies reflecting the positioning of early endosomes, what would the authors predict regarding the positioning of late endosomes (or multivesicular bodies).
* How do Golgi stressors impact on protein secretion ?
* Does the relocalization of FAM129A, impact on protein secretion ? and in particular does it promote a change in the balance between conventional and unconventional protein secretion
* The schematic representation of the model the authors are pushing forward is not very informative. Obtaining data on protein secretion would definitely help the authors to further document this aspect.

Reviewer 2

Advance summary and potential significance to field

The manuscript by Simon et al from the Hellerschmied lab investigates the impact of stress-induced changes at the Golgi apparatus. They used nigericin, which was previously used to induce Golgi stress and performed proteomics. The main finding is that Golgi stress results in a depletion of Golgi transport proteins, which they propose to be a shared, early phenotype.
This manuscript, although interesting feels rather preliminary. However, because this is a submission for a short article, I think the work is suitable for the scope of this journal and the "short article" category. A better understanding of Golgi stress will certainly be of interest for the community working on organelles and secretory trafficking. I have the following comments:

- I think the authors need to show that the induction of FAM129A is indeed ATF4 dependent.
- If ATF4 plays a role, why are the authors ruling out that FAM129A induction is mediated by the a Golgi stress response and not by the UPR or the integrated stress response?
- I think that the part of the manuscript that proposes a role for Golgi stress in melanoma is rather under-developed (even for a short article). In Fig3D there is not even a statistical significance. The authors might want to add more data to strengthen this part of the manuscript.

First revision

Author response to reviewers' comments

Reviewer 1: In this manuscript submitted as a short report to the Journal of Cell Science and titled « Stress-induced proteome remodeling at the Golgi-endosome interface », Simon and colleagues describe the impact of various stress conditions on the remodeling of the proteome at the interface between the Golgi complex and endosomes.

The manuscript is well written and experimentally sound. Data are of great interest and value for the community. The authors document the molecular events occuring upon stress in the Golgi apparatus and for that use a proteomics approach combined with the use of select stress inducing drugs. One can regret that the functional impact of such stress response (through protein relocalization) on protein secretion is not interrogated, especially regarding the central dogma of the manuscript which is that stress (and Golgi stress) induces the remodeling of the secretory pathway.

© 2026. Published by The Company of Biologists under the terms of the Creative Commons Attribution License (https://creativecommons.org/licenses/by/4.0/).

Comments

* Most if not all endosomal stainings are performed using anti EEA1 antibodies reflecting the positioning of early endosomes, what would the authors predict regarding the positioning of late endosomes (or multivesicular bodies).

R1A) We found that Golgi stress induced by nigericin or OSBP-targeting compounds induces changes in the Golgi and post-Golgi compartments. When characterizing the Golgi endosome-interface we focused in this study on the marker EEA1. However, we predict that also late endosomes will be affected by the rearrangements in response to stress. In our APEX2-proximity labeling dataset, we observed RAB7A as enriched in the proximity of FAM129A upon nigericin treatment. This suggests localization of FAM129A also to late endosomes, providing the position and composition of late endosomes during Golgi stress as a matter to explore in future work.

* How do Golgi stressors impact on protein secretion?

R1B) To address this point, we analyzed the effect of nigericin, on the transport of a plasma membrane protein through the secretory pathway. We performed live cell imaging studies of secretory pathway transport using the Retention Using Seletive Hooks (RUSH) system. We found that nigericin specifically impairs RUSH cargo exit from the Golgi apparatus. These new data are shown in **Figure 1B**, **Movie S1 and S2** and described in the text as follows:

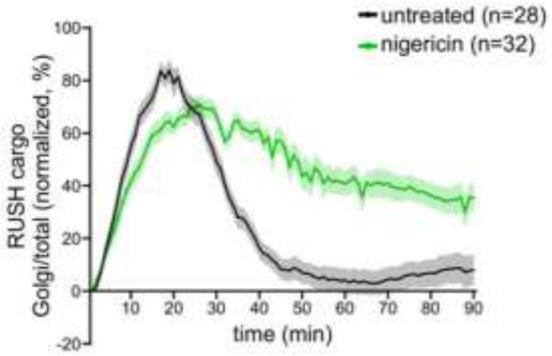

***Figure 1B**. RUSH assay kinetics of HeLa cells expressing Str-li-IRES-VSVG-SBP-EGFP and ST6Gal1-mCherry. Cells were treated with 1 µM nigericin 4 h prior to biotin addition or left untreated.Quantitative data depicts combined mean ± SEM of single cells for each condition. Data obtained from two independent experiments.*
*To understand how nigericin-induced Golgi stress impacts anterograde trafficking through the Golgi, we monitored transport kinetics using the Retention Using Selective Hooks (RUSH) system (Boncompain et al 2012) (**Fig. 1B, Movie S1** and **S2**). The plasma membrane-targeted RUSH cargo VSVG-SBP-GFP is retained at the ER by Ii-Str and released by addition of biotin. Under control conditions, the RUSH cargo reached the Golgi, as defined by the marker ST6Gal1-mCherry within 15-22 min of biotin addition. By 50 min after biotin addition, the majority of the cargo has passed through the Golgi (**Fig. 1B**). Pre-treatment of the cells with nigericin for 4 h caused a delay in export from the Golgi, where even after 90 min of biotin addition, a large fraction of the RUSH cargo still localized to the Golgi (**Fig. 1B**).*

* Does the relocalization of FAM129A, impact on protein secretion? and in particular does it promote a change in the balance between conventional and unconventional protein secretion

R1C) We would like to thank the reviewer for raising this interesting question. To address the impact of nigericin-induced relocalization of FAM129A on protein secretion, we combined the RUSH assay, as described above, with the expression of the stress response target FAM129A. Specifically, we expressed FAM129A^{RFP} in HeLa cells and applied nigericin for 4 h to induce an enrichment of FAM129A at the secretory pathway. We then followed the transport of the RUSH cargo VSVG-SBP-EGFP through the Golgi apparatus. In these experiments, we did not find FAM129A to impact the cellular secretory capacity. These data are now included in **Figure 3F** and described in the text as

© 2026. Published by The Company of Biologists under the terms of the Creative Commons Attribution License (https://creativecommons.org/licenses/by/4.0/).

follows:

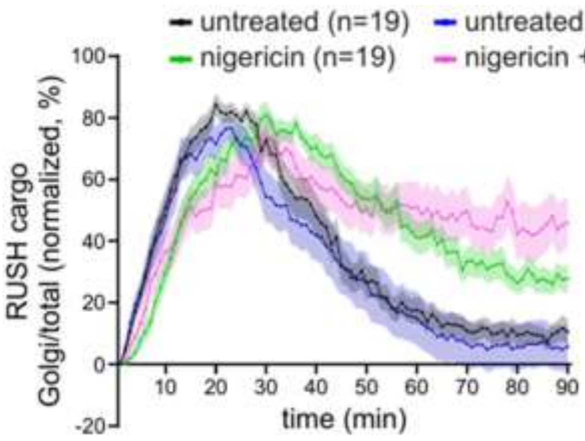

Figure 3F. *RUSH assay kinetics of HeLa Kyoto cells expressing Str-li-IRES-VSVG-SBP-EGFP and GRASP65-SNAP +/-FAM129A^{RFP}. Cells were pre-treated with 1 µM nigericin for 4 h prior to biotin addition or left untreated. Quantitative data depicts combined mean ± SEM of single cells for each condition. All cells recorded in one experiment.*

*We then explored whether FAM129A would affect protein secretion using the RUSH assay. We found that the expression of FAM129A^{RFP} and its nigericin-induced change in localization did not affect the secretory capacity of the cell (**Fig. 3F**).*

In preparing the revised manuscript, we furthermore explored whether Golgi stress activates unconventional secretion, as previously described for starvation-induced stress by Nuechel et al 2021 (doi: 10.1016/j.molcel.2021.06.017). To do so, we analyzed the dephosphorylation of the Golgi matrix protein GRASP55 on threonine 264 as a marker for the activation of unconventional protein secretion, as established by Nuechel et al 2021 (**Figure Rev1**). We first confirmed the dephosphorylation of GRASP55 T264 upon rapamycin and torin treatment, mimicking starvation conditions (**Figure Rev1AB**). In contrast, nigericin treatment did not induce changes in the phosphorylation state of GRASP55 T264. The effectiveness of the nigericin treatment was confirmed by the decrease of NUCB1 and SDF4. The total levels of GRASP55 were not affected by either of the applied treatments (**Figure Rev1B**).

Our data indicate that the nigericin-induced Golgi stress does not induce a shift towards unconventional protein secretion. We would however like to add that our analysis of a published data set indicates that it may be interesting to explore a link between FAM129A and unconventional secretion under starvation-induced stress in future studies. A proximity labeling experiment using GRASP55-APEX2 (Nuechel et al. 2021, Table S1) suggests proximity of the bait to FAM129A in starved cells. This research line however is beyond the scope of the current short report and we have therefore not included these data in the manuscript, but focused our analysis on conventional secretion and stress-induced remodeling of the secretory pathway.

NOTE: We have removed unpublished data that had been provided for the referees in confidence.

* The schematic representation of the model the authors are pushing forward is not very informative. Obtaining data on protein secretion would definitely help the authors to further document this aspect.

We have updated our model including the following findings obtained during the revision process (now presented in **Figure 4H**):

- Golgi export of secretory cargo along the conventional secretion route is impaired in response to nigericin.
- In response to stress, FAM129A is enriched on TGN46- and EEA1-positive carriers at the remodeled Golgi-endosome interface, but does not contribute to protein secretion.

© 2026. Published by The Company of Biologists under the terms of the Creative Commons Attribution License (https://creativecommons.org/licenses/by/4.0/).

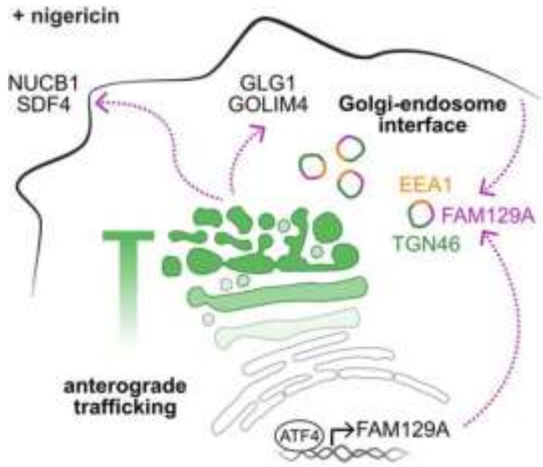

Figure 4H. Model of stress-induced remodeling of the secretory pathway.

Reviewer 2: The manuscript by Simon et al from the Hellerschmied lab investigates the impact of stress-induced changes at the Golgi apparatus. They used nigericin, which was previously used to induce Golgi stress and performed proteomics. The main finding is that Golgi stress results in a depletion of Golgi transport proteins, which they propose to be a shared, early phenotype.

This manuscript, although interesting feels rather preliminary. However, because this is a submission for a short article, I think the work is suitable for the scope of this journal and the "short article" category. A better understanding of Golgi stress will certainly be of interest for the community working on organelles and secretory trafficking. I have the following comments:

- I think the authors need to show that the induction of FAM129A is indeed ATF4 dependent.

R2A) To study the stress-induced expression of FAM129A in more detail we turned to melanoma cells. In the differentiated melanoma cell line Ma-Mel-61a, we showed an upregulation of FAM129A in response to nigericin and tunicamycin, in line with our data obtained from HeLa and RPE1 cells. We then generated a Ma-Mel-61a ATF4 knock-out cell line and found that the stress-induced expression of FAM129A is strongly impaired in this ATF4 depletion model. These data are shown in **Figure 4B** and **S4B** and described in the text as follows:

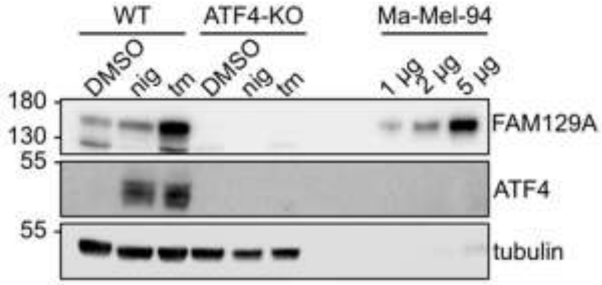

Figure 4B*. Western blot analysis of FAM129A and ATF4 in Ma-Mel-61a wild-type (WT) and ATF4 knock-out (KO) cells treated with 1 µM nigericin or 5 µg/ml tunicamycin for 18 h (25 µg applied). Ma-Mel-94 lysate applied for comparison.*

In the differentiated melanoma cell line Ma-Mel-61a with comparatively low basal FAM129A levels, we observed that FAM129A expression was induced in response to Golgi and ER stress (**Fig. 4B, S4B**). Using a corresponding Ma-Mel-61a ATF4 knock-out (KO) cell line, we found that this stress-induced FAM129A expression is largely dependent on ATF4 (**Fig. 4B, S4B**).

- If ATF4 plays a role, why are the authors ruling out that FAM129A induction is mediated by a

© 2026. Published by The Company of Biologists under the terms of the Creative Commons Attribution License (https://creativecommons.org/licenses/by/4.0/).

Golgi stress response and not by the UPR or the integrated stress response?

R2B) We thank the reviewer for this question. We did not mean to exclude that FAM129A expression is induced by the UPR or ISR. In this short report, we corroborated that the stress-induced expression of FAM129A is largely ATF4-dependent. How ATF4 is induced downstream of Golgi stressors is still an open question. We therefore did not mean to make any conclusions regarding this aspect and the revised manuscript we refer to a *transcriptional stress response* without reference to a transcriptional *Golgi-specific* stress response. The part of the manuscript now reads as follows:

*In addition to the depletion of Golgi transport proteins, nigericin induces a transcriptional stress response (Serebrenik et al 2018), including several target genes of the transcription factor ATF4. Consistently, nigericin induces the expression of ATF4 in HeLa cells, following 4 h of treatment. The kinetics of ATF4 expression are slower than with the established ER stressor thapsigargin, but show a comparable level of induction (**Fig. S2A**). To understand how the transcriptional stress response relates to the remodeling of the secretory pathway, we focused on FAM129A an ATF4 target gene, induced by nigericin, proteotoxic Golgi stress, but also ER stress (Pallmann et al 2019, Serebrenik et al 2018).*

- I think that the part of the manuscript that proposes a role for Golgi stress in melanoma is rather under-developed (even for a short article). In Fig3D there is not even a statistical significance. The authors might want to add more data to strengthen this part of the manuscript.

R2C) To address this point, we expanded our data on the stress response protein FAM129A in the context of the melanoma disease model. We characterized the stress-induced expression of FAM129A and its constitutive expression in dedifferentiated melanoma cells. While the stress-induced expression largely depends on ATF4 (see also R2A), the constitutive expression cannot be explained by the ATF4 expression state. We demonstrate constitutively high levels of FAM129A at the proteome level (Figure 4A). To analyze the FAM129A expression pattern in a larger dataset, we relied on bulk mRNA expression data. These data showed higher FAM129A levels in dedifferentiated cells - observed in two datasets, with statistical significance in one of them, as pointed out by the reviewer. We have additionally analyzed patient-derived single cell RNA sequencing data. From these data we extracted the melanocytic (differentiated) cell state and the dedifferentiated mesenchymal-like cell state. These data corroborate higher FAM129A levels in the dedifferentiated state (**Fig. S4C**). Taken together our data establish FAM129A as overexpressed in dedifferentiated melanoma cells at the mRNA and protein level. Moreover, our work supports regulatory mechanisms beyond ATF4-dependent, stress-induced FAM129A expression in the context of a melanoma dedifferentiation program (**Fig. 4**).

Second decision letter

MS ID#: jcs.264535R1

MS Title: Stress-induced proteome remodeling at the Golgi-endosome interface

Authors: Doris Hellerschmied; Gina Simon; Savina Abraham-Pol; Melisa Dendusic; Nina Schulze; Simone Stupia; Michelle Koci; Lana Buzuk; Beatrice Thier; Philine Steinbach; Hai Trinh; Jasmin Schillinger; Sabrina Ninck; Farnusch Kaschani; Markus Kaiser; Michael Ehrmann; Annette Paschen

Article Type: Short Report

Dear Dr Hellerschmied,

I am happy to tell you that your manuscript has been accepted for publication in Journal of Cell Science, pending standard publication integrity checks.

© 2026. Published by The Company of Biologists under the terms of the Creative Commons Attribution License (https://creativecommons.org/licenses/by/4.0/).
